# Developing an understanding of sophorolipid synthesis through application of a central composite design model

**DOI:** 10.1111/1751-7915.14003

**Published:** 2022-01-17

**Authors:** Benjamin Ingham, James Winterburn

**Affiliations:** ^1^ Department of Chemical Engineering and Analytical Science The University of Manchester Oxford Road Manchester M13 9PL UK

## Abstract

A key barrier to market penetration for sophorolipid biosurfactants is the ability to improve productivity and utilize alternative feedstocks to reduce the cost of production. To do this, a suitable screening tool is required that is able to model the interactions between media components and alter conditions to maximize productivity. In the following work, a central composite design is applied to analyse the effects of altering glucose, rapeseed oil, corn steep liquor and ammonium sulphate concentrations on sophorolipid production with *Starmerella bombicola* ATCC 222144 after 168 h. Sophorolipid production was analysed using standard least squares regression and the findings related to the growth (OD_600_) and broth conditions (glucose, glycerol and oil concentration). An optimum media composition was found that was capable of producing 39.5 g l^–1^ sophorolipid. Nitrogen and rapeseed oil sources were found to be significant, linked to their role in growth and substrate supply respectively. Glucose did not demonstrate a significant effect on production despite its importance to biosynthesis and its depletion in the broth within 96 h, instead being replaced by glycerol (via triglyceride breakdown) as the hydrophilic carbon source at the point of glucose depletion. A large dataset was obtained, and a regression model with applications towards substrate screening and process optimisation developed.

## Graphical abstract

By applying a central composite circumscribed design of experiment, it has been possible to model the contribution of media components towards sophorolipid production. Through this, an optimised control has been developed alongside a predictive tool with applications in feedstock screening.

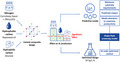

## Introduction

Chemical surfactants are essential to our modern life, appearing in some form in nearly every market sector, with applications in medicine, food, industrial processing, cosmetics, personal care and home care. Derived from petroleum, these surfactants are being increasingly scrutinized due to their persistence and detrimental effect on the environment and wildlife (Langberg *et al*., 2019; Kaczerewska *et al*., 2020). Biosurfactants are an eco‐friendly, non‐toxic and biodegradable alternative produced by microorganisms that have potential to disrupt the surfactant market, which has forecasted growth of up to 52.4 billion USD by 2025 (MarketsandMarkets, 2020). Sophorolipid biosurfactants present the greatest potential for market entry due to the high levels of productivity that can be gained from the wild‐type strain *S. bombicola* ATCC 22214, reaching levels > 300 g l^–1^ (Rau *et al*., 2001). A glycolipid biosurfactant, sophorolipids are composed of disaccharide sophoroses (2‐O‐β‐d‐glucopyranosyl‐d‐glucopyranose) linked to a hydrophobic terminal/sub‐terminal hydroxylated fatty acid group via a glycosidic bond (Nuñez *et al*., 2001).

Despite this, market entry is hindered by the high costs of production attributed to the cost of food‐grade feedstocks (Ashby *et al*., 2013). Furthermore, food‐crop‐derived feeds directly compete with the agricultural industry, causing greater economic instability to the areas where crops are grown (Hertel *et al*., 2012; Winchester and Reilly, 2015; Gerbens‐Leenes, 2018). Primarily, sophorolipid production is reliant on a nitrogen source (for cell growth), a hydrophilic carbon (for production of the sophorose monomers) and a hydrophobic carbon (for production of the fatty acid group). As such, there is a need to select alternative feedstocks rich in the required nutrients that are low cost and do not compete with food crops (for land use/direct consumption). The high number of potential feedstocks quickly becomes prohibitive to screen in bioreactors, so most are typically screened at shake flask scale. Whilst overall production is reduced due to limitations in mass transfer and reduced control of fermentation conditions, shake flask screening provides an excellent directional tool for identifying potential feedstocks. This approach has been applied to the screening of numerous potential feedstocks including waste frying oils, non‐feed crop oils and hydrolysed biomass (Fleurackers, 2006; Wadekar *et al*., 2012; Kaur *et al*., 2019; Marcelino *et al*., 2019).

However, when attempting to screen feedstocks there is a poor understanding within the literature over the maximum potential productivity that can be gained at a given scale/model that can be used as an internal control for which to compare against. Whilst some papers apply controls with food‐grade feedstocks to compare against, they typically do not demonstrate that their control is at an optimum for that scale. By demonstrating the maximum productivity of a given scale through a ‘best‐case’ control, the efficacy of a potential feedstock can easily be assessed. In some cases, no controls are applied, instead opting to refer to other works where different scales/media compositions and controls have been applied, which reduces the ability to identify whether any beneficial effect on production is caused by the feed or the difference in scale/process. Similarly, alternative feedstocks are often replaced ‘like‐for‐like’ with their food‐grade counterpart in media that may have been optimized for production despite differences in composition (nutrients, fatty acids, etc.), quality and the presence of potential inhibitors. An understanding of the media components in a fermentation media, their purpose and effect on productivity and potential interactions is essential to effectively screen a potential feedstock. By understanding this, the media composition can be altered to bolster the production potential of a feedstock and avoid it being tested in sub‐optimal conditions that may mask its use.

The application of statistical design of experiments provides an understanding of media component effects by creating structured experimental conditions where media components (factors) can be varied by concentration (levels) to generate a design region/space where the changes in productivity (output) can be analysed. Response surface methodologies (RSM) dictate a specific design that models the relationship between factors and the output once a statistical model is applied to estimate linear, quadratic and cubic curvature and two‐factor interactions. As such, the aim of this work was to apply an RSM to test the composition of a simple fermentation media for sophorolipid production, targeting the hydrophobic and hydrophilic carbon and nitrogen sources. A variety of RSMs can be applied for this purpose; however, a central composite circumscribed design (CCCD) was chosen due to its low prediction variance and efficient estimation of linear and quadratic interactions, which allow more accurate profiling of each factor (Witek‐Krowiak *et al*., 2014; Jankovic *et al*., 2021). In order to relate the findings of the statistical model to the underlying biological function of *S. bombicola* ATCC 22214, broth conditions were monitored and the changes observed (substrate consumption, cell mass and productivity) related to the profiles found in the regression model.

In order to select components and gain an understanding of suitable conditions for high SL production, a literature review was performed. Table 1 highlights the results of the literature search, identifying works that used high‐quality/food‐grade feedstocks in a batch/fed‐batch model with no influence from external production modifications (i.e. gravity separation). The high levels of production gained by Rau *et al*. (2001) and Davila *et al*. (1992) led to the selection of glucose, rapeseed oil, corn steep liquor (CSL) and ammonium sulphate for application in the model. From this, the CCCD design was applied and model subsequently developed to determine the key components for SL production, with analysis of the fermentation broth to relate changes in composition (glucose, glycerol and rapeseed oil) to the production achieved after 168 h incubation.

**Table 1:** Overview of selected papers with productive SL processes.

	Dolman *et al*. (2017)	Rau *et al*. (2001)	Shah *et al*. (2007)	Davila *et al*. (1992)	Casas and García‐Ochoa (1999)
Hydrophilic carbon source	100 g l^–1^ glucose	100 g l^–1^ glucose	100 g l^–1^ glucose	100 g l^–1^ glucose	100 g l^–1^ glucose
Hydrophobic carbon source	50 g l^–1^ rapeseed oil	100 g l^–1^ (50% C18:1) rapeseed oil, refined	40 g l^–1^ oleic acid	100 g l^–1^ rapeseed oil ethyl ester	100 g l^–1^ sunflower oil
Nitrogen source	6 g l^–1^ yeast extract, 5 g l^–1^ peptone	4 g l^–1^ (NH_4_)2SO_4_, 5 g l^–1^ CSL	10 g l^–1^ yeast extract, 10 g l^–1^ urea	4 g l^–1^ (NH_4_)2SO_4_, 5 g l^–1^ CSL	1 g l^–1^ yeast extract
Inoculation ratio (% v/v)	10	5	10	10	10
Conditions	25°C fed batch	25°C fed batch	30°C fed batch	24°C fed batch pH stat	30°C fed batch
Production (g l day^–1^)	25.68	57	4.2	40.42	15

## Results

Using a central composite circumscribed design model, a series of 50 ml fermentation flasks were incubated over a 168 h period to determine the effect of glucose, rapeseed oil and CSL/ammonium sulphate on SL production. The following section describes the initial findings and the iterative process that the model was taken through, exploring the initial central composite model, augmentation and subsequent improved regression with a wider dataset. In addition, at‐line samples were taken and quantified to understand the growth and the metabolic consumption profile of *S. bombicola*. The full data set with actual and predicted values can be found in Table S1.

### Design 1 – Overview of production

**Fig. 1:**
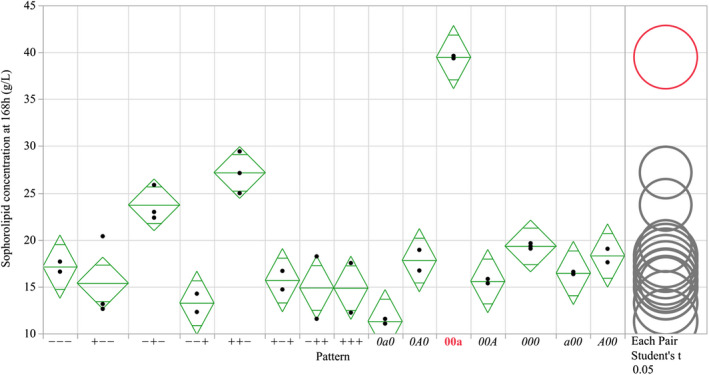
Sophorolipid produced at harvest (168 h) in flasks with differing levels of glucose, oil and nitrogen in Design 1 of the central composite model. Mean diamonds represent the 95% confidence interval (diamond tips), group mean (central line) and overlap mark (top and bottom insets) of each group. The least significant difference (α = 0.05) is represented graphically with comparison circles, with groups that are significantly similar (red) or different (grey) from the selected group (00a) shown.

As shown in Fig. 1, the applied levels of glucose, oil and nitrogen in the factorial and axial ranges of Design 1 were capable of producing a range of sophorolipid quantities at harvest from 11.3 to 39.5 g l^–1^ SL. The centrepoint (‘000’) combination, based on literature concentrations of each media component, was capable of producing a mean of 19.3 g l^–1^ sophorolipid. Amongst the three media components, alteration of the nitrogen had the greatest effect to production. By changing the nitrogen concentration from 5 to 0.8 g l^–1^ CSL and retaining the centrepoint values for glucose and oil, the fermentation was able to reach 39.5 g l^–1^ SL (‘00a’), producing a significantly different value from all other tested combinations in the design (highlighted by the comparison circles).

Alteration of the quantity of rapeseed oil in the media also had a discernible effect on SL production. As oil was reduced below 50 ml l^–1^ (‘‐’ of the pattern), production began to decline, with axial values (15.9 g l^–1^ oil, 0a0) only capable of producing 11.3 g l^–1^ SL. Inversely, by altering levels from 50 to 100 g l^–1^ rapeseed oil (‘‐‐‐’ to ‘‐+‐’), it was possible to show a statistically significant increase in production from 17.1 to 23.7 g l^–1^ SL (*P* = < 0.05).

Glucose did not show statistically significant differences to SL production in the ranges tested in Design 1 (15.9–184 g l^–1^). Whilst there appears to be a boost in production between the combination ‘‐+‐’ and ‘++‐’ from 23.7 to 27.2 g l^–1^, this is not statistically significant (*P* = < 0.05).

### Design 1 – Regression analysis

**Fig. 2:**
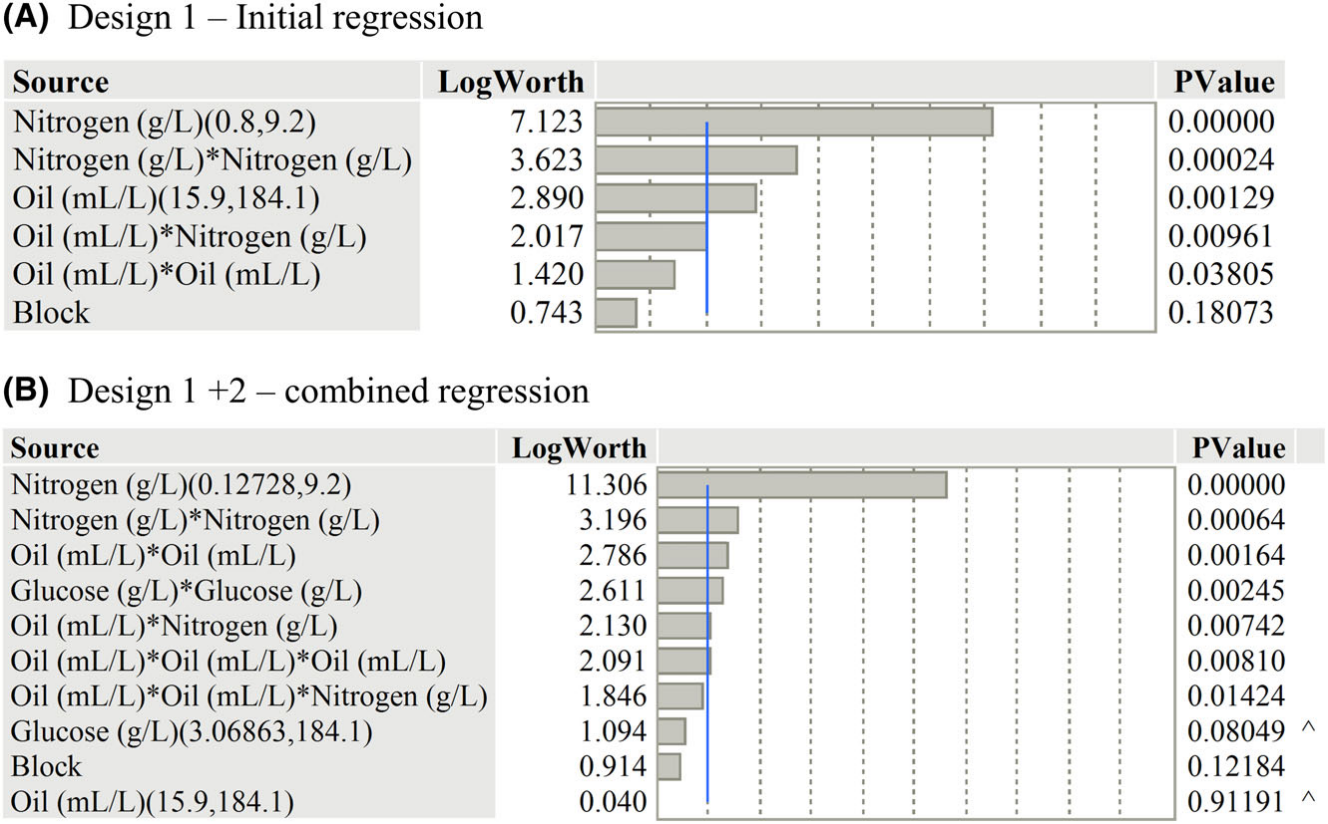
Pareto plot of selected model terms after stepwise regression to find significant factors in (A) Design 1 and (B) Design 2 of the central composite model. Major terms in the model have LogWorth values > 2.0 (indicated by the blue line). Regression is performed on each model term within the concentration limits stated on the linear terms (brackets: Lower limit, Upper limit). Retained non‐significant lower‐order terms are indicated by ‘^’.

Following completion of the fermentation flasks of Design 1, the final SL concentrations were inputted into JMP and linear, quadratic and linear interaction terms selected through stepwise regression for the term combination with the lowest BIC value. For details on the regression equation and model fit, see Figs S1‐S3. Following this, standard least squares regression was performed on the terms shown in Fig. 2A. The standard least squares regression demonstrated an *R* ^2^ adjusted value of 0.77 and root‐mean‐square error of 3.29 g l^–1^ SL. The high production value of ‘00a’ (40 g l^–1^) was marked as an outlier in studentized residual (exceeding 95% individual t‐limits) and actual/predicted plots (see Figs S2‐S3). As shown in the plot (Fig. 2A), nitrogen and oil both had a significant effect on the production of sophorolipids, demonstrating quadratic curvature and an interaction between the two terms.

The results of Design 1 indicate that the glucose range tested did not demonstrate a significant effect on the final sophorolipid concentration. From the literature, it has been shown that there should be a point where production begins to decline as the glucose concentration is reduced. As such, it was assumed that the concentration range initially tested was not capable of producing this effect, requiring further reduction to find the minimum substrate requirements.

A boost in SL production was observed when the nitrogen concentration was reduced to the axial value (0.8 g l^–1^ CSL, 0.64 g l^–1^ ammonium sulphate). This value was the lowest concentration of nitrogen tested. As such, it would be impossible to conclude that the nitrogen concentration found in Design 1 was truly optimum for SL production. Furthermore, this concentration of nitrogen was only tested against a single concentration of glucose and oil (pattern ‘00a’), with no indication of how variation of these two components would alter SL production. As such, it was decided that a second central composite design (Design 2) would be performed, reducing the glucose and nitrogen concentrations further to augment the current data set and apply further regression with higher‐order terms (cubic curvature and quadratic‐linear interactions).

### Design 2 – Overview of production

**Fig. 3:**
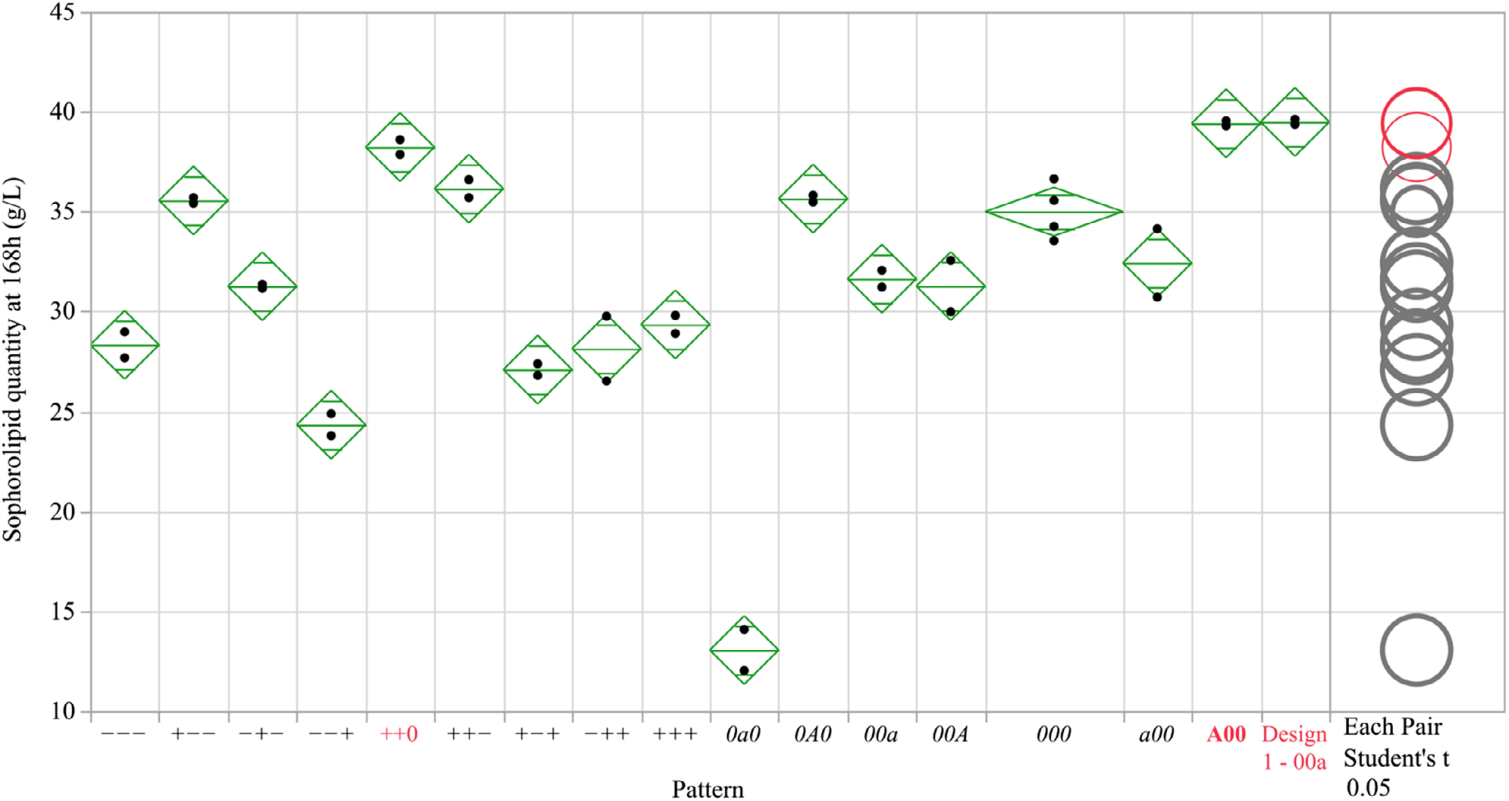
Sophorolipid produced at harvest (168 h) in flasks with differing levels of glucose, oil and nitrogen in Design 2 of the central composite model. Mean diamonds represent the 95% confidence interval (diamond tips), group mean (central line) and overlap mark (top and bottom triangles) of each group. The least significant difference (α = 0.05) is represented graphically with comparison circles, with groups that are significantly similar (red) or different (grey) from the selected group (Design 1 – 00a) shown.

Following augmentation of the central composite design, a series of flasks were run to explore a range of reduced glucose (3.07–61.93 g l^–1^) and nitrogen concentrations (0.1273–1.4727 g l^–1^ CSL, 0.102–1.178 g l^–1^ ammonium sulphate) in the hope of further maximizing SL production and determining the concentrations at which SL production would begin to decline for each media component. The results of the Design 2 flasks are shown in Fig. 3. Overall, Design 2 flasks show a higher level of SL production than Design 1, with the majority exceeding 27 g l^–1^ SL, up to a maximum of 39.42 g l^–1^ (‘A00’). Of the combinations tested, two demonstrate statistically comparable values (as shown by comparison circles, *P* = < 0.05) to the optimum flask combination found in Design 1 (‘00a’), with ‘++0’ and ‘A00’ with an average of 38.23 and 39.42 g l^–1^ SL respectively.

Similar to Design 1, the alteration of nitrogen had the greatest impact on production, with factorial flasks with higher concentrations of nitrogen (patterns ‘‐‐+’, ‘+‐+’, ‘‐++’ and ‘+++’) demonstrating statistically lower SL production (Student’s *t*‐test, *P* = < 0.05) compared to their counterparts with reduced levels (e.g. ‘‐‐+’ to ‘‐‐‐’ and ‘+‐+’ to ‘+‐‐’). Flasks with rapeseed oil at 50 ml l^–1^ (‘‐’ of the pattern) did see some reduction in the production of SLs; however, in general the levels of SL production in these flasks were higher than those in Design 1, where 50 ml l^–1^ oil was used, ranging from 24.33 g/L (‘‐‐‐’) to 35.56 g/L (‘+‐‐’) SL. At axial levels of oil (15.9 ml l^–1^), only 13.1 g l^–1^ SL was produced.

Unlike Design 1, the range of glucose tested in Design 2 (3.07–61.93 g l^–1^) was such that it was possible to observe a statistically significant decline (Student’s *t*‐test, *P* = < 0.05) in SL production when glucose levels were reduced (excluding in patterns ‘+++’ to ‘‐++’). However, even with 3.07 g l^–1^ glucose (‘a00’) flasks were still capable of producing an average of 32.43 g l^–1^ SL.

### Combined design regression

The combination of data from Design 1 and 2 provides 69 SL production values from 34 unique combinations of glucose, rapeseed oil and CSL/ammonium sulphate, to which a regression model can be applied. With this larger data set, higher‐order terms were included in the regression (compared with the data set from Design 1 only) to determine the significance of cubic curvature and quadratic‐linear interactions and to improve the predictive model. For details on the regression equation and model fit, see Figs S4‐S6 and Table S2. The selected significant model terms, following stepwise regression, are shown in the Pareto plot in Fig. 2B. As shown in Table 2, the combined regression model produced showed an improved adjusted *R* ^2^ and RMSE from the regression of Design 1, with the previous outlier (Design 1, ‘00a’) sitting along the predicted/actual regression line and within the confidence limits of the studentized residuals (see Fig. S6). Two patterns, Design 1 ‘+‐‐’ and Design 2 ‘00a’, were possible outliers in the regression as they exceeded 95% individual t‐limits. In addition, replicate variance was different between the two (Design 1 ‘+‐‐’, σ = 4.33 g l^–1^, Design 2 ‘00a’, σ = 0.6 g l^–1^ SL); however, as neither exceed Bonferroni residual limits (95% simultaneous) they were retained in the regression.

**Table 2:** Summary of fit for the regression models from the Design 1 and combined Design 1/2 data set. Design 1 + 2 includes the addition of cubic and quadratic‐linear interactions.

	Design 1 – initial regression	Design 1 + 2 – combined regression
$R^{2}$	0.813786	0.899597
$R_{\text{Adj}}^{2}$	0.772405	0.877691
Root‐mean‐square error	3.28629	3.140124
Mean of response	19.01794	25.15309
Observations	34	68

The augmented regression model retained identical terms to Design 1, namely in the significance, curvature and interaction between the oil and nitrogen terms. The increased ranges tested in the model for nitrogen and glucose did lead to some changes in the significance. The linear term of nitrogen has an increased LogWorth value from 7.123 (Design 1) to 11.306 (Design 1 and 2 combined), whilst glucose was found to present quadratic curvature over the tested range. Whilst the linear term for oil is of low significance in Design 2, this is because its significance is contained within the cubic curvature (Oil*Oil*Oil) term. However, glucose still did not demonstrate statistical significance towards the production of sophorolipids, being retained in the model due to the significance of the quadratic term. The addition of new terms in the regression identified cubic curvature for rapeseed oil, with the addition of a quadratic‐linear interaction between the oil and nitrogen (Oil*Oil*Nitrogen).

**Fig. 4:**
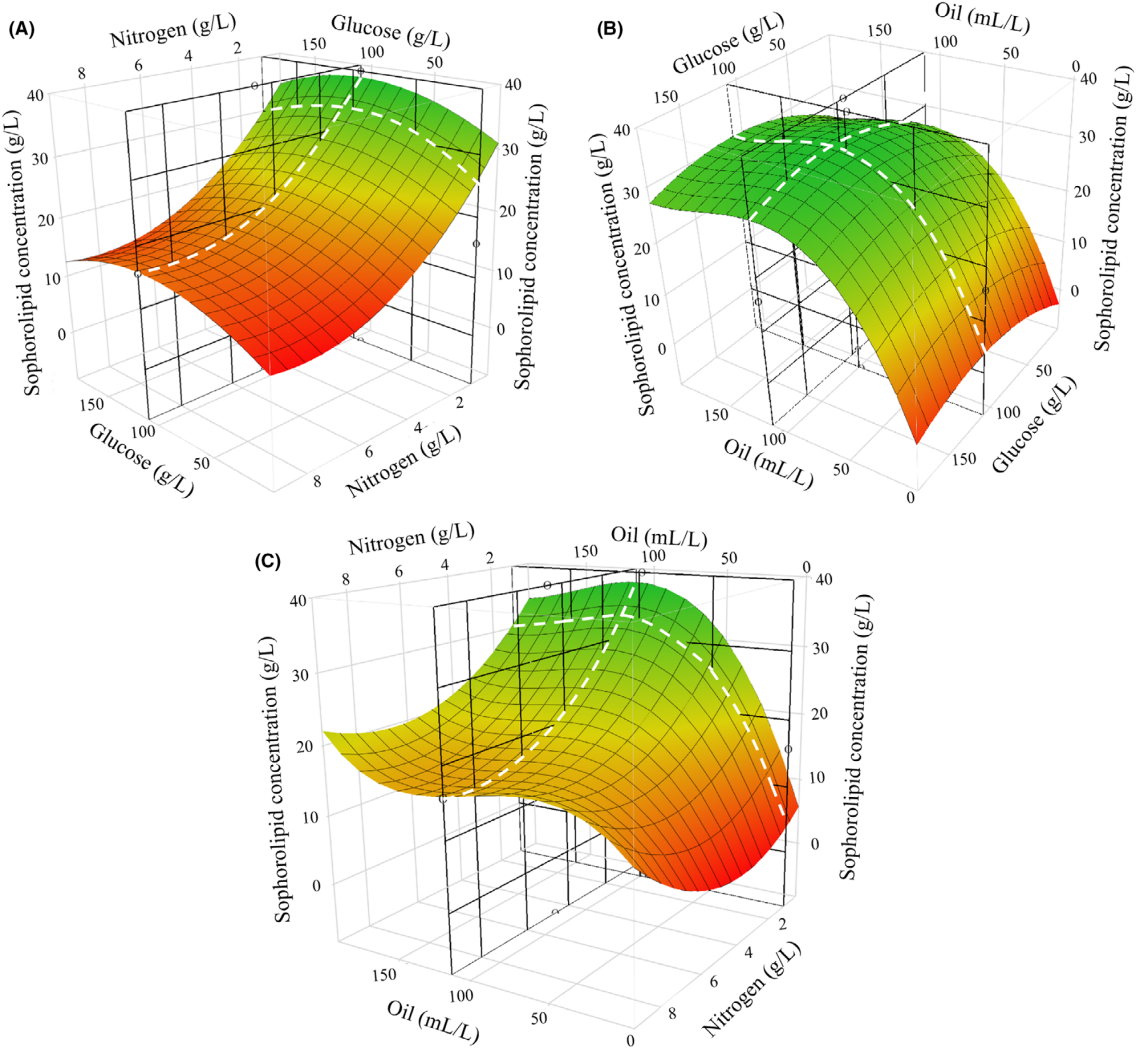
3D surface plots comparing model terms (A) glucose and nitrogen (B) glucose and oil and (C) oil and nitrogen using the predictive formula produced following model regression. Surface plots are coloured from low (red) to high (green) final sophorolipid concentration at 168 h, with a mesh applied on the surface to show 3D topography. Predictive modelling was used to set each term to its predicted optimum value, as shown by the intersecting grid lines (black) The surface curvature at these optimum points (white dashes) is representative of the profile for each media component in the predictive model.

Surface plots were generated to demonstrate the interaction between any two of the media components with their effect on the final sophorolipid concentration at 168 h shown in a 3D space. Figure 4 shows the effects of altering the concentration of two given media components on SL production. As shown, alteration of the components changes the shape and contour of the surface (with respect to the final sophorolipid concentration), allowing for an ‘optimum point’ to be found, as marked by the intersecting lines on the surface plots. Looking at the individual effect of each component, the plots demonstrate that SL production increases as nitrogen is lowered, up to the limits tested in the model (0.13 g l^–1^ CSL, 0.104 g l^–1^ ammonium sulphate). Conversely, a sharp rise in SL production is seen as oil concentration increases, up to an optimum point close to the centre of the tested range (112 ml l^–1^). Glucose demonstrates a similar upward trend towards an optimal point (108 g l^–1^); however, the effect of moving from the least (3.06 g l^–1^) and most optimum (108 g l^–1^) concentration has a lower effect on production (33.38 g l^–1^ to 40 g l SL) compared with oil.

As shown in the model output/Pareto plot, glucose did not interact with the other terms of the model. This is similarly reflected in Fig. 4A and B, where the contour of the surface (shown on the surface mesh) along the glucose axis retains its profile (a quadratic curvature), regardless of the nitrogen and oil concentration. Similarly, the same relationship is shown when nitrogen and oil are compared against changing glucose concentrations, retaining their curvature (quadratic and cubic respectively). It should be noted that whilst the cubic profile of oil is not pronounced in Fig. 4B, this is due to the nitrogen concentration being set to the predicted optimum (0.127 g l^–1^), which reduces the amount of curvature. This curvature is more pronounced at higher levels of nitrogen (8–10 g l^–1^) in Fig. 4C on the surface plots, with the surface profile of the oil surface changing as the nitrogen concentration is altered. Similarly, the nitrogen demonstrates a sharp quadratic curvature upwards as concentrations reduce when oil concentrations are > 100 g l^–1^; however, this curvature becomes much shallower as the oil concentration is reduced.

**Fig. 5:**
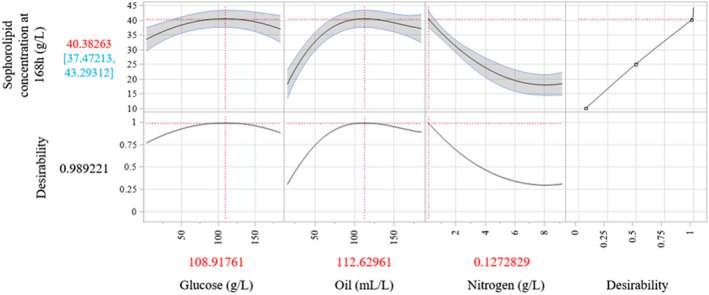
Predictive model output from standard least squares regression of the selected significant terms in the combined data from Design 1 and 2 of the central composite model. Term values at the bottom are those predicted to provide maximum sophorolipid quantities at 168 h.

The predictive model generated from the regression model was able to determine the optimum concentrations of glucose, oil and nitrogen that were capable of producing a theoretical sophorolipid concentration of 40 g l^–1^ (Fig. 5). This model provides a 2D interpretation of the surface profile of each individual component as their concentration is adjusted (with the other 2 components maintained at the optimum concentration), matching the highlighted surfaces on Fig. 4 (shown by the white dashes).

### Interaction between media components and sophorolipid production

**Fig. 6:**
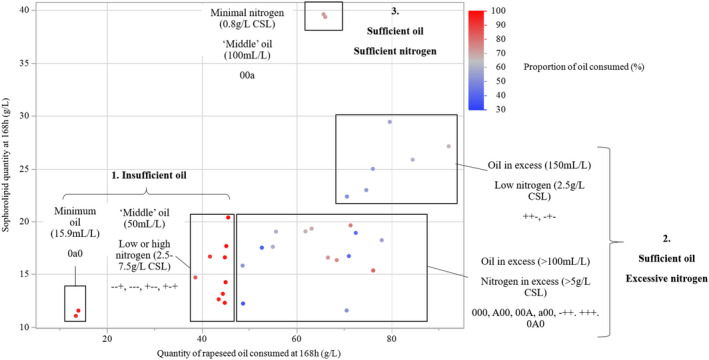
Relationship between the quantity and relative proportion of rapeseed oil consumed in the fermentation media to the quantity of sophorolipid at harvest (168 h) from fermentations in Design 1 of the central composite model. Selected points are grouped based on their initial nitrogen (relative to CSL) and oil concentrations.

Alongside final sophorolipid concentration, residual rapeseed oil at harvest was quantified and used to determine the amount of oil consumed. Figure 6 demonstrates the relationship between the rapeseed oil consumption of flasks in Design 1 and their final sophorolipid concentration. As shown, flasks with low levels of oil (< 50 ml l^–1^) show no residual oil at harvest (100% consumed) regardless of nitrogen concentration (Group 1), with all of them demonstrating low sophorolipid production (< 20 g l^–1^ SL). As the oil concentration is increased (> 100 ml l^–1^), none of the flasks fully consume the available oil (excess oil) and the final sophorolipid concentration increases as the nitrogen level is reduced from 5 g l^–1^ CSL (Group 2, bottom square), to 2.5 g l^–1^ CSL (Group 2, top square) until it finally reaches 0.8 g l^–1^ CSL where peak sophorolipid production is seen.

### Influence of media components on growth and media composition

**Fig. 7:**
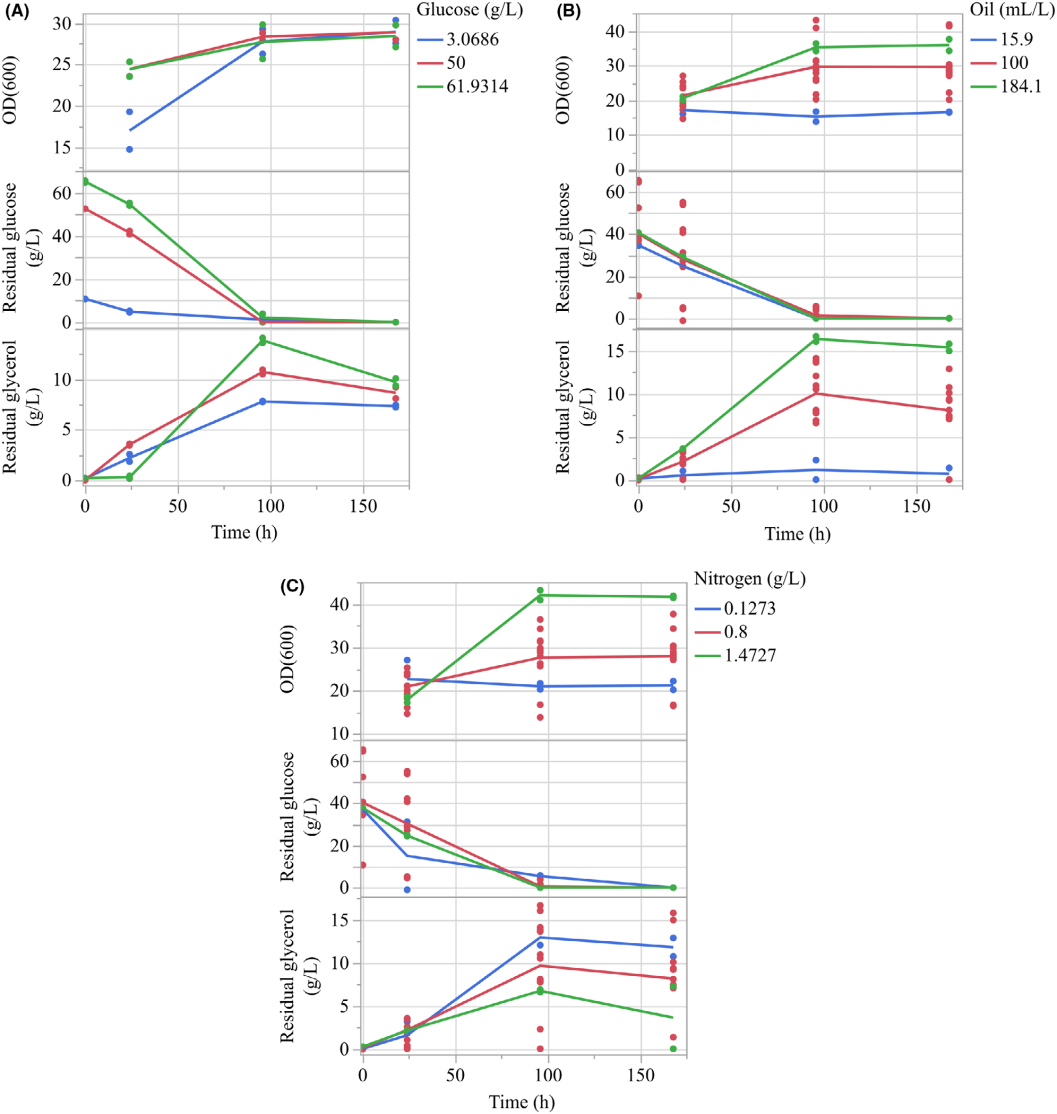
Influence of initial (A) glucose, (B) oil and (C) nitrogen/CSL concentration in the fermentation media on the growth (OD_600_), glucose consumption and generation of glycerol over a 168 h fermentation period.

In order to link the statistical trends seen in the regression model to the actual conditions within the fermentation broth growth (OD_600_), glucose and glycerol were quantified in selected flasks over the 168 h period. Initial quantification of optical density was hindered by overestimation and high variance caused by components of the fermentation broth. The addition of an ethanol cleaning step led to a quantification method with low variability that was applied to the axial block (all flasks tested containing axial ‘A’ or ‘a’ values) of Design 2. As such, this block was chosen to analyse growth, with the additional quantification. The effect of glucose, nitrogen and oil on the optical density, glucose and glycerol profiles of the sophorolipid flasks is shown in Fig. 7, with quantification at 0, 24, 96 and 168 h. In general, all flasks demonstrated the same consumption/production profile for glucose and glycerol. Initially, small amounts of glucose were consumed between 0 and 24 h, at which point the consumption rate increased until glucose was fully depleted by 96 h. During this period, the glycerol concentration increased until glucose became fully depleted, at which point the glycerol began to be consumed as the concentration declined for the remainder of the fermentation. In all flasks, glycerol never reached 0 g l^–1^ during the 168 h fermentation period.

Initial glucose concentration (Fig. 7A) does not effect the growth of *S. bombicola* in the fermentation broth, with the exclusion of one group (3.07 g l^–1^ glucose) that, whilst lower initially, is able to match the growth levels of the other flasks by 96 h. Logically, increased initial concentrations of glucose lead to greater levels in the broth at 24 h. By 96 h, glucose is fully consumed regardless of initial concentration. Similarly, the higher initial levels of glucose lead to a higher level of glycerol in the broth up until the point of glucose depletion is reached.

As shown in Fig. 7B, varying the starting oil concentration does not initially cause a change in growth (24 h); however, growth begins to diverge from 96 h, with higher levels of oil leading to higher OD_600_ values. Glucose levels are not influenced by changes in oil concentrations. However, higher levels of rapeseed oil lead to greater levels of residual glycerol in the fermentation broth up until the point of glucose depletion by 96 h. Following this, glycerol consumption occurs at the same rate regardless of initial oil concentration (as shown by gradient between 96 and 168 h: −0.012, −0.019 and −0.012 for 15.9, 100 and 184.1 ml l^–1^ respectively).

Whilst initial growth (24 h) does not show statistically different OD_600_ values (Student’s *t*‐test, *P* = < 0.05), increases in initial nitrogen (Fig. 7C) lead to increased growth by 96 h, which is sustained to the point of harvest (168 h). Whilst initially similar (24 h), glycerol generation separates by 96 h, with higher residual concentration in flasks with lower initial nitrogen. Glycerol consumption is similar between 0.8 and 1.4 g l^–1^ between 96 and 168 h (gradient = −0.033 and −0.04, respectively), but far lower at 0.127 g l^–1^ nitrogen/CSL (gradient = −0.05). Residual glucose levels are initially similar (0–24 h) between flasks of different initial nitrogen, with 0.8 and 1.4 g l^–1^ CSL/nitrogen both reaching depletion by 96 h. However, flasks with 0.127 g l^–1^ nitrogen still demonstrate residual glucose at 96 h.

## Discussion

The aim of this work was to develop a small‐scale model of SL production that would better the understanding of the SL production process in relation to the media components and changes within the fermentation broth. Through the application of a CCCD response surface methodology, an optimized media composition could be found and a predictive model developed, with future applications in feedstock screening, tracking individual component influence and how they interact together in order to hinder/boost production.

### Design rationale

A major design decision was to simplify the composition of the fermentation media to the three core constituents for sophorolipid biosynthesis, guided by the current understanding in order to easily link changes in production to a specific component. Nitrogen is essential in stimulating the growth phase of the fermentation, allowing for the accretion of SL producing cells, but there is a trade‐off between gaining a higher cell density and the increased length of the growth phase. In order to generate the sugar moiety of the sophorolipid structure, UDP‐glucose must be generated via glycolysis/glycogenesis, driven by a hydrophilic carbon source. Whilst more complex sources can be used, glucose provides the most direct/simple precursor to the glycolysis pathway, leading to a more productive SL fermentation (Van Bogaert *et al*., 2008; Rispoli *et al*., 2010; Bhangale *et al*., 2014; Konishi *et al*., 2016). The generation of the SL tail via terminal or sub‐terminal hydroxylation of C16‐C18 length fatty acids can be supplied directly (free fatty acids) or through lipid precursors (triglycerides) and has been noted to be the most vital part in gaining a productive process (Davila *et al*., 1994; Cavalero and Cooper, 2003; Felse *et al*., 2007). The high oleic acid content of rapeseed oil (61.6% in this work) and its relatively low cost (particularly in comparison with pure fatty acids) made it an obvious choice (Daverey and Pakshirajan, 2009; Van Bogaert *et al*., 2010). The supply of oil must be well controlled as it can easily cause changes in the broth rheology, affecting oxygen mass transfer and ultimately leading to a decline in production, particularly at shake flask scale, where agitation is reliant solely on shaking of the broth. By simplifying to three media components with distinct purposes in SL synthesis, the effects they have on SL production can easily be identified avoiding confounding from other/more complex media components.

### Applying central composite designs to maximize production of SL

Through the application of an iterative central composite circumscribed design of experiments, a fermentation media composition was found that was capable of achieving 40 g l^–1^ SL in 168 h at 50 ml scale using only four media components. This gives confidence that the maximum potential SL production has been found at this shake flask scale (50 ml working volume) with these 3 media components, providing a ‘best‐case’ internal control to compare against when substituting with a feedstock during screening.

This is a good level of production from *S. bombicola* ATCC 222144 when comparing against other works of similar scale (shake flask), quantification method (ethyl acetate/gravimetric) and feedstock type (food grade/high purity). At 50 ml Erlenmeyer scale, Daverey and Pakshirajan (2009) were able to achieve 45 g l^–1^ in 192 h with a soybean oil and sugarcane molasses media whilst Shah *et al*. (2017) reached 32 g l^–1^ in 120 h with palm oil, glucose, yeast extract and urea. With the inclusion of pure oleic acid (the preferred carbon‐length fatty acid for SL synthesis), authors see a major increase in productivity, from 55.2 g l^–1^ (192 h, 50 ml scale) to 95.4 g l^–1^ (144 h, 10 ml scale) (Kurtzman *et al*., 2010; Jadhav *et al*., 2019). Productivity is improved as the fatty acids are supplied directly to the cells, rather than through indirect fatty acid precursors, such as triglycerides, alkanes and fatty acid ethyl/methyl esters. The performance of the fermentation in this work is benefited by the procedural media optimisation and the inclusion of baffled flasks to improve mixing and mass transfer. The oxygenation of the fermentation broth was a major point of consideration for this SL fermentation, as the biphasic (water/oil) media and viscous properties of the product are known to cause a decline in mass transfer and impede productivity (Saerens *et al*., 2015; Dolman *et al*., 2017).

### Applications of the data set and predictive capabilities

One of the key challenges to market entry for sophorolipids is the high cost of feedstocks, meaning alternative, low‐cost sources derived from non‐food crops and agricultural by‐products are required (Ashby *et al*., 2013). The sheer quantity of potential feedstocks that can be screened, at a range of concentrations and combinations, is generally prohibitive to test at bioreactor scale. As such, screening must be performed at shake flask scale¸ opting to forgo the beneficial process control (pH, temp, aeration and stirring) that increases sophorolipid productivity. With this in mind, it is important to understand the limitations of the shake flask scale model that is being applied, developing an internal control that demonstrates the maximum productivity that can be achieved at that scale. Without an understanding of the minimum and maximum range of productivity of a model, the potential of a feedstock may be masked. Unfortunately, many works within the literature do not apply suitable controls or instead opt to compare against other authors that have different scales/models. The work here distinguishes itself from the literature by having a proven, robust ‘best‐case’ model that can be used to compare feedstocks and provide a between‐run control.

The large amount of data produced has also provided a robust predictive model that provides an understanding of the effects of reducing/increasing the three primary media components. Ultimately, this model can be applied to screening; by understanding the composition of a given feedstock in relation to its glucose, nitrogen and fatty acid content, it will be possible to alter the composition of the fermentation media, guided by the predictive model, to maximize production. The model developed is capable of showing statistically significant changes in SL production as components are altered, with values from 11.06 to 40 g l^–1^ SL, which gives confidence that any detrimental/beneficial changes to the fermentation process will likely cause a visible change in production.

As shown, the regression model possesses statistical strength (adjusted *R* ^2^ = 0.878), with a low level of potential outliers. Those outliers that were identified indicate that further advancements could be made to both the regression model and the quantification. From Design 1 (Fig. 1), it was clear that the gravimetric quantification method had a greater level of variability at lower concentrations of SL at the 5 ml harvest quantity. Whilst larger quantities at harvest can help to resolve this, the application of more accurate quantification methods such as HPLC and LC‐MS would reduce variability, improve accuracy and ultimately improve the predictive capabilities of the model (Davila *et al*., 1993; Ratsep and Shah, 2009).

Standard least squares regression is a powerful tool for approximating models, but can encounter limitations when handling complex non‐linear functions over a large range, such as those found in the quadratic‐linear interactions of the rapeseed oil and nitrogen. To determine accuracy of the current model, extra shake flask experiments were performed after this series of work using the predicted optimum media composition (Fig. 5) which was only capable of producing an average of 33 g l^–1^ SL, rather than the 40 g l^–1^ predicted. Given the breadth of concentrations tested in this work, further investigation should be applied to look at advanced regression models capable of more accurately predicting SL production, exploring potential points within the design space where extra experimental data could improve the accuracy of predictions.

### Effect of nitrogen on productivity and growth

As described, nitrogen may be supplied to the fermentation media in order to stimulate the growth of cells, enabling a higher density of cells capable of producing SL. In order to enter SL production, cells must go through a stress state, which is presumed to be caused by the depletion of nitrogen. This entry into the production phase is demonstrated by the distinction in growth after 24 h (Fig. 7C), where lower levels of nitrogen (0.1273 and 0.8 g l^–1^ CSL) see little increase in cell mass as nitrogen depletes and they begin to enter the production phase of the fermentation. At higher levels (1.47 g l^–1^ CSL), cell mass increases greatly between 24 and 96 h as there is a greater availability of nitrogen in the media which increases the length of the growth phase, delaying entry to the production phase. This is seen in the quadratic curvature of nitrogen (Fig. 4). Initially, the tested range of nitrogen (10–3 g l^–1^ CSL) is too high to allow entry into the productive state. As levels reduce (3–0.127 g l^–1^), nitrogen is more likely to become fully depleted, allowing the cells to become productive, with greater production at lower levels of nitrogen. It is important to note, however, that whilst the regression model indicates 0.127 g l^–1^ CSL as optimum, the actual best tested concentration was 0.8 g l^–1^ CSL, suggesting some level of nitrogen is required to provide a small amount of growth that bolsters production.

Within the literature, there is conflicting information on the importance of nitrogen for SL production. The application of models/design of experiments, such as those of Minucelli *et al*. (2017) and Rispoli *et al*. (2010), indicates that the presence of nitrogen is generally prohibitive to SL production. Conversely, papers with the highest level of productivity (used to formulate the initial media composition for this work) use far greater levels of nitrogen than those found in this work (5 g l^–1^ CSL, 4 g l^–1^ ammonium sulphate or 6 g l^–1^ yeast extract, 5 g l^–1^ peptone) (Davila *et al*., 1992; Rau *et al*., 2001; Dolman *et al*., 2017). Both Rau *et al*. (2001) and Davila *et al*. (1992) are able to show complete consumption of nitrogen within the early stages of the fermentation, suggesting that the improved scale (shake flask to bioreactor) and mode of operation (batch to fed batch) allows for greater consumption of nitrogen. Despite this, the findings of this work still apply; the levels of nitrogen must be optimized to gain a balance between sufficient growth and a lengthy production phase in order to be productive. At larger scale, the exact nitrogen requirements may alter; however, the fundamental balance of growth/production still remains.

### Effect of rapeseed oil on productivity and growth

Following model regression, it was shown that rapeseed oil has an important role in sophorolipid production (Fig. 2B). This is in good agreement with the literature, where removal or decline of hydrophobic carbon supply (via triglycerides, ethyl esters or other fatty acid residues) results in a large decline in production (Davila *et al*., 1992; Rau *et al*., 1996). Within the ranges tested, there was a clear decline in production as oil levels declined < 50 ml l^–1^, indicating that the minimum concentration for production had been found. This is supported by the relation between sophorolipid yield, oil consumption and initial oil concentrations (Fig. 6); as levels decline < 50 ml l^–1^, there is insufficient oil to sustain production over the 168 h period and final sophorolipid concentrations decline. Minimum oil requirements have not been extensively studied in batch models within the literature; however, fed‐batch models have shown maximum production with consumption of 140 g l^–1^rapeseed oil or 184 g l^–1^ rapeseed ethyl ester as examples (Davila *et al*., 1992; Rau *et al*., 2001). As well as improving SL production, increased levels of oil appear to increase biomass (Fig. 7B). Whilst the exact reason for this is unclear, there has been demonstration of increased biomass by association of fatty acids to large vacuoles in *S. bombicola* that could lead to alteration of the cell size and subsequent optical density (Hommel *et al*., 1994).

As shown in the surface (Fig. 4) and predictive (Fig. 5) plots, SL production rapidly increases from 50 ml l^–1^ of rapeseed oil until it begins to flatten as it reaches the optimum value, indicating that the levels of oil become sufficient to sustain production. As the oil concentration increases past the optimum value, production begins to decline, indicating that higher levels of oil begin to inhibit the SL production. This has similarly been evidenced in the literature, where an excess of rapeseed oil, over 10 g l^–1^ in fed‐batch models, has been shown to cause a decline in production, presumably through the toxic effect of excess fatty acids and the impact on mass transfer (Rau *et al*., 1996). The presence of cubic curvature for oil is only exaggerated at higher levels of nitrogen in the surface plots/predictive models (Figs 4 or 6); however, this is more likely an over‐exaggeration by the regression model, particularly as combinations of high oil (> 150 ml l^–1^) and high nitrogen (> 5 g l^–1^ CSL) have not actually been tested, with the model instead assuming an increase in production based on two design points (Design 1 and 2, patterns 0A0).

### Interaction between oil and nitrogen

As shown in the regression model, significant interactions were found between the oil and nitrogen (Fig. 2B) and how they effected the subsequent production of SL. The two terms interact both in a linear relation (Oil*Nitrogen) and in relation between the curvature of oil and linear effect of nitrogen (Oil*Oil*Nitrogen). At its simplest, the two terms work to effectively inhibit the effect of the other depending on their initial concentration. Following Fig. 7C, a high initial nitrogen concentration will nullify the effect of altering the amount of oil on SL production, as the cells focus solely on growth and do not utilize rapeseed oil, leading to a ‘flattening’ of the profile of the oil. Inversely, as oil levels begin to decline, the beneficial effect of reducing nitrogen on SL production is reduced as the oil becomes fully depleted, removing the primary substrate for SL production from the fermentation media. These effects are shown directly in the fermentation broth (Fig. 7C), where greater levels of growth are seen with higher nitrogen (as the growth phase is sustained) and glycerol production is lowered, indicating that triglyceride breakdown (and general consumption of oil) is reduced.

The interaction is also reflected in Fig. 6. At lower levels of oil (< 50 ml l^–1^), the substrate supply for SL production is insufficient and nitrogen exerts no effect on improving SL production. As oil levels increase (> 100 ml l^–1^), the substrate supply becomes sufficient to sustain SL production for the 168 h period (as shown by oil consumption levels being < 100%). At this point, SL production is reliant on reducing the nitrogen concentration, as the fermentation becomes either focussed on production (low nitrogen) or growth (high nitrogen). The findings here highlight how one media component may exert an effect on another and must be carefully considered/modelled. In the case of oil and nitrogen, it is important to understand the core purpose of each component (i.e. substrate supply and biomass development) and consider how they may hinder/boost one another.

### Effect of glucose on productivity

Hydrophilic carbon sources, in particular glucose, are highlighted in the literature as being an important component in sophorolipid production, driving glycolysis and glycogenesis to generate UDP‐glucose, the precursor to the sophorose moiety of sophorolipids (Hommel *et al*., 1994). For example, whilst Davila *et al*. (1997) found that production could occur with the sole presence of rapeseed ethyl esters, the inclusion of glucose during the production phase significantly increased sophorolipid production. When comparing against other DoE work, the importance of glucose is repeated. Rispoli *et al*. (2010) found that sugar, even in disaccharide form (sucrose, fructose and lactose), was required to gain a productive process. Minucelli *et al*. (2017) applied a Box Benkhen design not too dissimilar from Design 2 at 25 ml working volume, exploring the optimum concentration of glucose, chicken fat and urea and found an 83% reduction in productivity when glucose concentrations were reduced from 100 to 10 g l^–1^. Comparatively, our work was able to produce an average 32.43 g l^–1^ SL even with only 3.086 g l^–1^ glucose (Design 2 – a00, Fig. 3).

Following the completion of Design 1, it was theorized that the original concentrations of glucose were all too high to lead to depletion within the broth, meaning a decline in SL production was not possible. However, consumption profiles in Design 2 (Fig. 7A–C) highlighted that glucose was actually depleted in most flasks by 96 h. With the assumption that the rate of consumption between sampling points (24 and 96 h) was identical, it would be expected that flasks with lower initial glucose would become depleted earlier and have a statistically significant decline in SL production if glucose was a significant term. Whilst there was evidence that increased glucose did improve production (32.43–39.42 g l^–1^ SL from patterns a00 to A00 of Design 2), presumably through its sustained presence in the broth, the level was not significant enough to be highlighted within the model.

### Interaction between glucose and glycerol

The likely explanation for this lack of significance can be found in the switch from glycerol accumulation (a by‐product of triglyceride breakdown for the generation of fatty acids) to consumption at the point of glucose depletion (Fig. 7A–C). Initially, *S. bombicola* is unable to co‐utilize glycerol in the presence of glucose due to carbon catabolite repression, leading to glycerol accumulation within the broth (Gancedo, 1998; Lin *et al*., 2019). At the point of glucose depletion, the repression is removed and glycerol becomes the new hydrophilic carbon source, sustaining SL production up to the point of harvest. As such, the presence of glycerol effectively masks the potentially deleterious effects of glucose depletion, leading to the lack of significance of glucose found in the model. Further to this, glycerol never fully depletes in the broth (even at the lowest oil concentration, 15.9 ml l^–1^, Fig. 7A), meaning there is no point at which a hydrophilic carbon source is not present. In reality, glucose and oil do interact; higher initial concentrations of glucose increase productivity as a more efficient hydrophilic carbon source is available for longer, whilst also generating glycerol. When glucose is eventually depleted, it requires a minimum level of oil to generate sufficient glycerol to continue the hydrophilic carbon source up to the point of harvest. It is likely that with longer fermentation periods or lower initial oil, glycerol would become fully depleted and the significance of glucose would have been highlighted.

The findings here indicate a potential balance that must be struck when selecting efficient hydrophilic carbon sources and glycerol‐producing hydrophobic sources. As sources rich in free fatty acids are expensive, it is more likely that oils rich in triglyceride forms of the required fatty acids will be used, leading to the production and accumulation of glycerol in the broth. Glycerol is known to exert an inhibitory effect on both upstream and downstream production of SLs as it effects broth rheology, mixing and separation (Buchholz *et al*., 1978; Lin *et al*., 2019). Within this work, the presence of glycerol caused complications in the downstream processing of the fermentation broth; glycerol levels > 5 g l^–1^ led to cell mass aggregation into distinct miniature ‘clumps’ that made isolation of the sophorolipid and oil phase difficult during solvent extraction. As a result, dry cell weight measurements were not possible in this work and subsequently led to the development of an ethanol treatment step to improve optical density measurements by removing glycerol from the broth. With this potential inhibitory effect in mind, there may be an advantage to allow for the depletion of an ‘efficient’ hydrophilic carbon source (i.e. glucose) temporarily to allow for the consumption of glycerol to levels where it does not exert an inhibitory effect. Fed‐batch production processes could easily monitor glycerol levels and alter the feeding rate of glucose to allow for glycerol consumption to remove this effect. However, this will be a balance between the potential decline in productivity from swapping to a less efficient hydrophilic carbon (glucose to glycerol) against the loss in productivity caused by glycerol accumulation.

## Conclusion

Through the application of a CCCD response surface methodology, a large data set capable of demonstrating the effect of varying three primary media components towards sophorolipid production has been produced. With the application of stepwise and standard least squares regression modelling, it has been possible to produce a statistically significant regression model that can profile these media components and find an optimum composition for SL productivity, with future applications in applying the predictive model in feedstock screening. An iterative design process, in which two of the model terms were augmented to supplement the data set, has been applied making it possible to explore a large design space and gain confidence that the optimal conditions have been found to maximize SL productivity at shake flask scale, giving a meaningful baseline SL productivity to measure feedstock performance against.

By monitoring the conditions within the fermentation broth, the output of the regression model has been linked to the changes seen within the fermentation and expands the understanding of the role of each component within sophorolipid fermentation. The importance of nitrogen was highlighted as high levels cause a sustained growth phase, reducing SL productivity. It will be interesting to see whether the requirements for nitrogen differ as the process is scaled up and fermentation control (pH, feed control and oxygen) improves, as there is mixed evidence in the literature of nitrogen requirements. Rapeseed oil is important as the primary substrate for SL production and was capable of minimizing the effect of glucose depletion within the broth, via the production (and subsequent consumption) of glycerol.

Despite its prominence in the literature, glucose did not have a significant effect on SL production, even though a wide range of concentrations were tested. The utilization of glycerol, generated via rapeseed oil metabolism, reduced any deleterious effect of glucose depletion, highlighting the potential of this by‐product to aid SL production. Controlled consumption of glycerol during SL fermentation through fed‐batch processes may help to supplement the hydrophilic carbon supply and reduce the presence of an inhibitory component, improving productivity.

## Experimental procedures

### Design 1 – Initial central composite circumscribed design

JMP 15 was used to generate an experimental design for a response surface model capable of studying the effect of glucose, rapeseed oil and corn steep liquor/ammonium sulphate on SL production, as shown in Table 3A. A central composite circumscribed design was chosen due to its suitability for process optimisation, with the assumption that non‐observed/controlled variables (temperature, trace elements and seed concentration) were consistent and unlikely to produce a statistically significant effect. For a CCCD, the media components are altered to different levels at low (‐), centre (0) and high (+) values (‘factorial’ range) initially, with the addition of extremely high (‘A’) and low (‘a’) values (‘axial’ range). To begin, the literature concentrations of glucose, rapeseed oil and corn steep liquor/ammonium sulphate from Rau *et al*. (2001) and Davila *et al*. (1992) were chosen as the centrepoints (000) within the design, with the factorial range chosen as +/− 50% of the centrepoint concentration (‘+’ and ‘‐’). The design was circumscribed with axial values (‘a’ and ‘A’) at an alpha value of 1.682, allowing for rotatability and consistent predictive variance from all points around the centrepoint. Corn steep liquor and ammonium sulphate were kept within the same ratio (5 g l^–1^:4 g l^–1^, respectively) as the nitrogen source development was not the focus of this work. All patterns were run in duplicate to determine variance and model fit. The decision was made to retain all factorial runs in one block and axial runs in another. This way any blocking effect (a change in the variance or values of any given replicate/repeat) would only influence the axial or factorial values respectively. An additional set of three patterns tested in block 1 (+‐‐, ++‐ and ‐+‐) were repeated once in block 2.

**Table 3:** Media composition of (A) Design 1 and (B) Design 2 of the central composite model. Each feedstock was tested at five concentrations, marked by symbols representing their position in the factorial (−, 0 and +) and axial (a and A) levels of the design.

A)						
		Concentration				
Component	Feedstock	a	−	0	+	A
Hydrophilic carbon	Glucose (g l^–1^)	15.9	**50**	100	150	184.1
Hydrophobic carbon	Rapeseed oil (ml l^–1^)	**15.9**	**50**	**100**	**150**	**184.1**
Nitrogen	Corn steep liquor (g l^–1^)	**0.8**	2.5	5	7.5	9.2
	Ammonium sulphate (g l^–1^)	0.64	2	4	6	7.36

B)					
	Concentration				
Feedstock	a	−	0	+	A

Glucose (g l^–1^)	3.07	15	32.5	**50**	61.93
Rapeseed oil (ml l^–1^)	**15.9**	**50**	**100**	**150**	**184.1**
Corn steep liquor (g l^–1^)	0.1273	0.4	**0.8**	1.2	1.4727
Ammonium sulphate (g l^–1^)	0.10184	0.32	0.64	0.96	1.17816

Values in bold are common tested concentrations between both designs.

### Design 2 – Augmentation of model

Following on from Design 1, it was decided that nitrogen and glucose concentrations needed further reduction to determine the effect on SL production. As such, the original design was augmented with a secondary central composite circumscribed design using JMP 15. The media component concentrations of Design 2 are summarized in Table 3B.

**Fig. 8:**
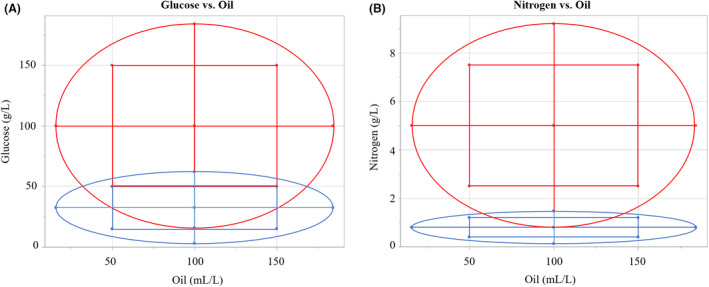
Relationship between Design 1 and 2 of the central composite design shown by the tested concentrations of (A) glucose and oil and (B) nitrogen and oil. The tested oil concentrations between both designs are identical. Each point represents the combination of factors tested in one fermentation flask, testing the factorial (square) or axial (circle) region of the design space. Overlap/shared points between the axial and factorial values of Design 1 and 2 are shown by the crossover of the two design spaces.

The reduction in these components aimed to serve two different purposes. For nitrogen, it was clear that high productivity was found around 0.8 g l^–1^ CSL, so this was placed as the centrepoint and a range of +/− 50% of that concentration used as the factorial values, with the hope of finding a precise optimum point. For glucose, there was indication that productivity did not decline between 50 (‘‐’ value) and 15.9 g l^–1^ glucose (‘a’ value), so the decision was made to explore this range to greater detail in the hope finding the point of decline, using the 50 g l^–1^as the upper factorial (‘+’) value and 15 g l^–1^ as the lower (close to the axial value tested in Design 1). As per the original design, axial values were added to obtain a fully rotatable design (alpha = 1.682) and values run in duplicate. The range of oil concentrations was retained from Design 1 as they were found to produce quadratic curvature (tailing at lower and higher concentrations), meaning the profile did not require further exploration. The relation between the two designs is shown graphically in Fig. 8. A total of 34 flasks were run and quantified for Design 2. An additional point (++0) was added to test a region of interest in the final block of Design 2.

### Chemicals and reagents

Stock solutions of glucose (Alfa Aesar, Heysham, UK), rapeseed oil (Crisp N’ Dry, Erith, UK) and CSL (Sigma Aldrich, St. Louis, MO, USA)/ammonium sulphate (Fluorochem Ltd, Hadfield, UK) were prepared and autoclaved separately at 121°C for 15 min.

### Microbial culture and maintenance

*Starmerella bombicola* ATCC 222144 was used in this study. Cryopreserved inoculum sourced from a single *S. bombicola* colony grown on YPG agar (6 g l^–1^ yeast extract, 5 g l^–1^ peptone and 100 g l^–1^ glucose) was prepared to inoculate seed culture flasks. A single colony was inoculated into media containing 5 g l^–1^ CSL, 4 g l^–1^ ammonium sulphate, 100 g l^–1^ rapeseed oil and 100 g l^–1^ glucose and incubated to mid‐exponential growth (OD_600_ = 10, 2.35 × 10^8^ cfu ml^–1^) prior to the addition of 10% glycerol and storage at −80°C.

### Production flask conditions

All cultures were performed in 250 ml 4‐bottom baffled flasks (Thermo Scientific Nalgene; Fisher Scientific, Loughborough, UK) with a 50 ml media fill volume, with the media composition being dictated by the model design. Flasks were incubated in an orbital shaker at 25°C, 200 rpm and 0.75 inch throw.

Production flasks were inoculated with 5 ml seed culture transferred at a target OD_600_ of 11–15.0 (mid‐exponential growth). Media used for the development of the seed consisted of 5 g l^–1^ CSL, 4 g l^–1^ ammonium sulphate, 100 g l^–1^ rapeseed oil and 100 g l^–1^ glucose (Rau *et al*., 2001).

### Analytical methods

Samples, 1 ml, were taken periodically in order to quantify the optical density and media composition of the fermentations. To prepare, 1 ml samples were centrifuged at 13 500 rpm for 5 min and supernatant removed for HPLC analysis. The cell pellet was then washed and vortexed with 0.5 ml 70% ethanol in order to remove residual sophorolipid and glycerol, followed by centrifugation at 13 500 rpm for 5 min, removal of the supernatant and resuspension of the pellet in 1 ml (original volume) 0.9% saline. This resuspension was used to quantify biomass via OD_600_ measurement using a UV‐spectrophotometer.

Glucose and glycerol quantification was performed with HPLC using an UltiMate® 3000 system with an Aminex HPX‐87P column and isocratic elution with 0.6 ml min^–1^ 5 mM H2SO4, with a 5 µL injection volume and column temperature of 50°C. Samples were analysed with a RefractoMax 520 refractive index detector (Thermo Fisher Scientific, UK) and compared with a calibration curve of glucose and glycerol. Glycerol was selected for quantification as it is a by‐product of the breakdown of triglycerides in the rapeseed oil required to generate the fatty acids for SL production, allowing for offline estimation of oil consumption in the fermentation broth.

### Liquid:liquid extraction

In order to gain a confluent sample for extraction and minimize phase separation within the broth, whole fermentation broths were transferred at harvest to 50 ml Falcon tubes and vortexed well (30 s, 2000 rpm), followed by immediate sampling of 5 ml whilst the broth was still agitated/in motion. Samples were then heated at 60°C for 15 min in a water bath in order to dissolve lactonic sophorolipid crystals.

Hexane extraction was used for rapeseed oil removal and quantification, followed by sophorolipid recovery and quantification using ethyl acetate. Equimolar quantities of hexane were added and vortexed at 2000 rpm for 15 s and the hexane phase removed onto a pre‐weighed aluminium tray 2 times, with the final extraction samples centrifuged at 5000 rpm for 10 min to ensure full removal of the hexane phase. To remove SL product, three separate ethyl acetate extractions were performed and dispensed on pre‐weighed aluminium trays. Both solvent extractions were allowed to dry overnight at ambient temperature on their pre‐weighed aluminium tray. These trays were then weighed and the final value calculated in relation to the final volume of broth. Quantities of rapeseed oil were converted from weight (g l^–1^) to volume (ml l^–1^) using the relative density of rapeseed oil (0.915) where required.?>

### Model regression

Following quantification of the sophorolipid for the tested fermentation flasks, regression analysis was performed to compare the effects of the hydrophilic carbon (glucose), hydrophobic carbon (rapeseed oil) and nitrogen (CSL/ammonium sulphate) factors on the final quantities of sophorolipids at harvest (168 h). Model effects were chosen to include single‐factor (X1), two‐factor (X1*X2) interactions and quadratic curvature (X1*X1). These were later supplemented in Design 2 with quadratic‐linear interactions (X1*X1*X2) and cubic curvature (X1*X1*X1).

Stepwise linear regression was performed on the JMP 15 platform to select a model with a combination of factors with the lowest Bayesian Information Criterion (BIC) score, with the inclusion of the blocking term regardless of significance. In cases where higher‐order terms (i.e. two‐factor, three‐factor and quadratic) demonstrated significance and lower terms (one‐factor) did not, the lower‐order terms were still retained in the regression. Following this, standard least squares regression was performed on the selected factors and analyses performed as detailed in this work.

## Code availability

All statistical analysis and modelling as performed using JMP 15.

## Consent to participate

Not applicable.

## Conflict of interest

There are no conflicts of interest with the authors of this work.

## Ethical approval

Not applicable.

## Authors’ contributions

BI was responsible for conceptualisation, execution and analysis of the experimental work, as well as the write‐up of the manuscript. JW was responsible for project supervision, project administration, funding acquisition as well as manuscript review and feedback.

## Supporting information


**Fig. S1**. Prediction expression for SL production with glucose, rapeseed oil and nitrogen (in relation to cornsteep liquor, with a ratio of 1:0.8g/L cornsteep liquor:ammonium sulfate) for the combined data set from Design 1. Model was sequentially developed from stepwise regression (for model effect combination with the lowest Bayesian Information Criterion value) and standard least squares regression.
**Fig. S2**. Predicted by actual plot of Design 1 values with the initial Design 1 regression model. Points labelled by pattern are those that are close to/exceed 95% individual t distribution limits as dictated by externally studentized residuals.
**Fig. S3**. Externally studentized residual plot of results from Design 1 with the initial regression model. Outer limits (red) are 95% Bonfonerri limits, and inner limits (green) are 95% individual t limits. Values close to/exceeding the inner limits are labelled by pattern.
**Fig. S4**. Prediction expression for SL production with glucose, rapeseed oil and nitrogen (in relation to cornsteep liquor, with a ratio of 1:0.8 cornsteep liquor:ammonium sulfate) for the combined data set from Design 1 and Design 2. Model was sequentially developed from stepwise regression (for model effect combination with the lowest Bayesian Information Criterion value) and standard least squares regression.
**Fig. S5**. Predicted by actual plot of Design 1 (red) and 2 (blue) of the CCD from JMP 15 with the combined regression model. Points labelled by pattern are those that are close to/exceed 95% individual t distribution limits as dictated by externally studentized residuals.
**Fig. S6**. Externally studentized residual plot of results from Design 1 (red) and Design 2 (blue) with the combined regression model. Outer limits (red) are 95% Bonfonerri limits, and inner limits (green) are 95% individual t limits. Values close to/exceeding the inner limits are labelled by pattern.
**Table S1**. Complete data set of executed fermentation flasks under Design 1 and 2 of the central composite circumscribed design. Actual sophorolipid quantities are supplied alongside the predicted values from the regression models of Design 1 and Design 1/2. Nitrogen values refer to the quantity of cornsteep liquor, with a ratio of 1:0.8g/L cornsteep liquor:ammonium sulfate.
**Table S2**. Analysis of variance of the combined regression model and model terms.

## Data Availability

The data that support the findings of this study are available from the corresponding author upon reasonable request.

## References

[mbt214003-bib-0001] Ashby, R.D. , McAloon, A.J. , Solaiman, D.K.Y. , Yee, W.C. , and Reed, M. (2013) A process model for approximating the production costs of the fermentative synthesis of sophorolipids. J Surfactants Deterg 16: 683–691.

[mbt214003-bib-0002] Bhangale, A. , Wadekar, S. , Kale, S. , Bhowmick, D. , and Pratap, A. (2014) Production of sophorolipids synthesized on castor oil with glucose and glycerol by using *Starmerella bombicola* (ATCC 22214). Eur J Lipid Sci Technol 116: 336–343.

[mbt214003-bib-0003] Buchholz, H. , Buchholz, R. , Niebeschütz, H. , and Schügerl, K. (1978) Absorption of oxygen in highly viscous newtonian and non‐Newtonian fermentation model media in bubble column bioreactors. Eur J Appl Microbiol Biotechnol 6: 115–126.

[mbt214003-bib-0004] Casas, J. , and García‐Ochoa, F. (1999) Sophorolipid production by *Candida bombicola*: medium composition and culture methods. J Biosci Bioeng 88: 488–494. 16232650 10.1016/s1389-1723(00)87664-1

[mbt214003-bib-0005] Cavalero, D.A. , and Cooper, D.G. (2003) The effect of medium composition on the structure and physical state of sophorolipids produced by *Candida bombicola* ATCC 22214. J Biotechnol 103: 31–41. 12770502 10.1016/s0168-1656(03)00067-1

[mbt214003-bib-0006] Daverey, A. , and Pakshirajan, K. (2009) Production, characterization, and properties of sophorolipids from the yeast *Candida bombicola* using a low‐cost fermentative medium. Appl Biochem Biotechnol 158: 663–674. 19082764 10.1007/s12010-008-8449-z

[mbt214003-bib-0007] Davila, A.‐M. , Marchal, R. , and Vandecasteele, J.‐P. (1992) Kinetics and balance of a fermentation free from product inhibition: sophorose lipid production by *Candida bombicola* . Appl Microbiol Biotechnol 38: 6–11.

[mbt214003-bib-0008] Davila, A.M. , Marchal, R. , Monin, N. , and Vandecasteele, J.P. (1993) Identification and determination of individual sophorolipids in fermentation products by gradient elution high‐performance liquid chromatography with evaporative light‐scattering detection. J Chromatogr 648: 139–149. 8245170 10.1016/0021-9673(93)83295-4

[mbt214003-bib-0009] Davila, A.‐M. , Marchal, R. , and Vandecasteele, J.‐P. (1994) Sophorose lipid production from lipidic precursors: predictive evaluation of industrial substrates. J Ind Microbiol 13: 249–257.

[mbt214003-bib-0010] Davila, A.M. , Marchal, R. , and Vandecasteele, J.P. (1997) Sophorose lipid fermentation with differentiated substrate supply for growth and production phases. Appl Microbiol Biotechnol 47: 496–501.

[mbt214003-bib-0011] Dolman, B.M. , Kaisermann, C. , Martin, P.J. , and Winterburn, J.B. (2017) Integrated sophorolipid production and gravity separation. Process Biochem 54: 162–171.

[mbt214003-bib-0012] Felse, P.A. , Shah, V. , Chan, J. , Rao, K.J. , and Gross, R.A. (2007) Sophorolipid biosynthesis by *Candida bombicola* from industrial fatty acid residues. Enzyme Microb Technol 40: 316–323.

[mbt214003-bib-0013] Fleurackers, S.J.J. (2006) On the use of waste frying oil in the synthesis of sophorolipids. Eur J Lipid Sci Technol 108: 5–12.

[mbt214003-bib-0014] Gancedo, J.M. (1998) Yeast carbon catabolite repression. Microbiol Mol Biol Rev 62: 334–361. 9618445 10.1128/mmbr.62.2.334-361.1998 PMC98918

[mbt214003-bib-0015] Gerbens‐Leenes, P.W. (2018) Green, blue and grey bioenergy water footprints, a comparison of feedstocks for bioenergy supply in 2040. Environ Process 5: 167–180.

[mbt214003-bib-0016] Hertel, T. , Steinbuks, J. , and Baldos, U. (2012) Competition for land in the global bioeconomy. Econ Agric, 44: 129–138.

[mbt214003-bib-0017] Hommel, R.K. , Weber, L. , Weiss, A. , Himmelreich, U. , Rilke, O. , and Kleber, H.P. (1994) Production of sophorose lipid by Candida (Torulopsis) apicola grown on glucose. J Biotechnol 33: 147–155.

[mbt214003-bib-0018] Jadhav, J.V. , Pratap, A.P. , and Kale, S.B. (2019) Evaluation of sunflower oil refinery waste as feedstock for production of sophorolipid. Process Biochem 78: 15–24.

[mbt214003-bib-0019] Jankovic, A. , Chaudhary, G. , and Goia, F. (2021) Designing the design of experiments (DOE) – An investigation on the influence of different factorial designs on the characterization of complex systems. Energy and Buildings 250: 111298.

[mbt214003-bib-0020] Kaczerewska, O. , Martins, R. , Figueiredo, J. , Loureiro, S. , and Tedim, J. (2020) Environmental behaviour and ecotoxicity of cationic surfactants towards marine organisms. J Hazardous Mat 392: 122299. 10.1016/j.jhazmat.2020.122299 32092649

[mbt214003-bib-0021] Kaur, G. , Wang, H. , To, M.H. , Roelants, S.L.K.W. , Soetaert, W. , and Lin, C.S.K. (2019) Efficient sophorolipids production using food waste. J Clean Prod 232: 1–11.

[mbt214003-bib-0022] Konishi, M. , Fujita, M. , Ishibane, Y. , Shimizu, Y. , Tsukiyama, Y. , and Ishida, M. (2016) Isolation of yeast candidates for efficient sophorolipids production: their production potentials associate to their lineage. Biosci Biotechnol Biochem 80: 2058–2064. 27251083 10.1080/09168451.2016.1191332

[mbt214003-bib-0023] Kurtzman, C.P. , Price, N.P.J. , Ray, K.J. , and Kuo, T.‐M. (2010) Production of sophorolipid biosurfactants by multiple species of the Starmerella (Candida) bombicola yeast clade. FEMS Microbiol Lett 311: 140–146. 20738402 10.1111/j.1574-6968.2010.02082.x

[mbt214003-bib-0024] Langberg, H.A. , Breedveld, G.D. , Grønning, H.M. , Kvennås, M. , Jenssen, B.M. , and Hale, S.E. (2019) Bioaccumulation of fluorotelomer sulfonates and perfluoroalkyl acids in marine organisms living in aqueous film‐forming foam impacted waters. Environ Sci Technol 53: 10951–10960. 31353899 10.1021/acs.est.9b00927

[mbt214003-bib-0025] Lin, Y. , Chen, Y. , Li, Q. , Tian, X. , and Chu, J. (2019) Rational high‐throughput screening system for high sophorolipids production in *Candida bombicola* by co‐utilizing glycerol and glucose capacity. Bioresour Bioprocess 6: 17.

[mbt214003-bib-0026] Marcelino, P.R.F. , Peres, G.F.D. , Terán‐Hilares, R. , Pagnocca, F.C. , Rosa, C.A. , Lacerda, T.M. , *et al*. (2019) Biosurfactants production by yeasts using sugarcane bagasse hemicellulosic hydrolysate as new sustainable alternative for lignocellulosic biorefineries. Ind Crops Prod 129: 212–223.

[mbt214003-bib-0027] MarketsandMarkets , (2020) Surfactants Market by Type (Anionic, Non‐Ionic, Cationic, and Amphoteric), Application (Home Care, Personal Care, Industrial & Institutional Cleaning, Textile, Elastomers & Plastics, Agrochemicals, and Food & Beverage), Region ‐ Global Forecast to 2025 [Market Report]. URL https://www.marketsandmarkets.com/Market‐Reports/biosurfactants‐market‐493.html

[mbt214003-bib-0028] Minucelli, T. , Ribeiro‐Viana, R.M. , Borsato, D. , Andrade, G. , Cely, M.V.T. , de Oliveira, M.R. , *et al*. (2017) Sophorolipids production by *Candida bombicola* ATCC 22214 and its potential application in soil bioremediation. Waste Biomass Valorization 8: 743–753.

[mbt214003-bib-0029] Nuñez, A. , Ashby, R. , Foglia, T. , and Solaiman, D. (2001) Analysis and characterization of sophorolipids by liquid chromatography with atmospheric pressure chemical ionization. Chromatographia 53: 673–677.

[mbt214003-bib-0030] Ratsep, P. , and Shah, V. (2009) Identification and quantification of sophorolipid analogs using ultra‐fast liquid chromatography–mass spectrometry. J Microbiol Methods 78: 354–356. 19559734 10.1016/j.mimet.2009.06.014

[mbt214003-bib-0031] Rau, U. , Hammen, S. , Heckmann, R. , Wray, V. , and Lang, S. (2001) Sophorolipids: a source for novel compounds. Ind Crops Prod 13: 85–92.

[mbt214003-bib-0032] Rau, U. , Manzke, C. , and Wagner, F. (1996) Influence of substrate supply on the production of sophorose lipids by *Candida bombicola* ATCC 22214. Biotechnol Lett 18: 149–154.

[mbt214003-bib-0033] Rispoli, F.J. , Badia, D. , and Shah, V. (2010) Optimization of the fermentation media for sophorolipid production from *Candida bombicola* ATCC 22214 using a simplex centroid design. Biotechnol Prog 26: 938–944. 20205261 10.1002/btpr.399

[mbt214003-bib-0034] Saerens, K.M.J. , Van Bogaert, I.N.A. , and Soetaert, W. (2015) Characterization of sophorolipid biosynthetic enzymes from Starmerella bombicola. FEMS Yeast Res 15: fov075. 26298016 10.1093/femsyr/fov075

[mbt214003-bib-0035] Shah, M.U.H. , Sivapragasam, M. , Moniruzzaman, M. , Talukder, M.M.R. , Yusup, S.B. , and Goto, M. (2017) Production of sophorolipids by *Starmerella bombicola* yeast using new hydrophobic substrates. Biochem Eng J 127: 60–67.

[mbt214003-bib-0036] Shah, V. , Jurjevic, M. , and Badia, D. (2007) Utilization of restaurant waste oil as a precursor for sophorolipid production. Biotechnol Prog 23: 512–515. 17286413 10.1021/bp0602909

[mbt214003-bib-0037] Van Bogaert, I.N.A. , De Maeseneire, S.L. , Develter, D. , Soetaert, W. , and Vandamme, E.J. (2008) Cloning and characterisation of the glyceraldehyde 3‐phosphate dehydrogenase gene of *Candida bombicola* and use of its promoter. J Ind Microbiol Biotechnol 35: 1085–1092. 18594888 10.1007/s10295-008-0386-x

[mbt214003-bib-0038] Van Bogaert, I.N. , Roelants, S. , Develter, D. , and Soetaert, W. (2010) Sophorolipid production by *Candida bombicola* on oils with a special fatty acid composition and their consequences on cell viability. Biotechnol Lett 32: 1509–1514. 20607361 10.1007/s10529-010-0323-8

[mbt214003-bib-0039] Wadekar, S. , Kale, S. , Lali, A. , Bhowmick, D. , and Pratap, A. (2012) Sophorolipid production by starmerella bombicola (ATCC 22214) from virgin and waste frying oils, and the effects of activated earth treatment of the waste oils. J Am Oil Chem Soc 89: 1029–1039.

[mbt214003-bib-0040] Winchester, N. , and Reilly, J.M. (2015) The feasibility, costs, and environmental implications of large‐scale biomass energy. Energy Econ 51: 188–203.

[mbt214003-bib-0041] Witek‐Krowiak, A. , Chojnacka, K. , Podstawczyk, D. , Dawiec, A. , and Pokomeda, K. (2014) Application of response surface methodology and artificial neural network methods in modelling and optimization of biosorption process. Biores Technol 160: 150–160. 10.1016/j.biortech.2014.01.021 24495798

