# Supplementary material for: Developing an understanding of sophorolipid synthesis through application of a central composite design model
Source: Microb Biotechnol. 2022 Jan 17;15(6):1744–61. doi: 10.1111/1751-7915.14003 (PMC9151336; doi:10.1111/1751-7915.14003)
Supplement: Supplementary file 1 — Fig. S1. Prediction expression for SL production with glucose, rapeseed oil and nitrogen (in relation to cornsteep liquor, with a ratio of 1:0.8g/L cornsteep liquor:ammonium sulfate) for the combined data set from Design 1. Model was sequentially developed from stepwise regression (for model effect combination with the lowest Bayesian Information Criterion value) and standard least squares regression. Fig. S2. Predicted by actual plot of Design 1 values with the initial Design 1 regression model. Points labelled by pattern are those that are close to/exceed 95% individual t distribution limits as dictated by externally studentized residuals. Fig. S3. Externally studentized residual plot of results from Design 1 with the initial regression model. Outer limits (red) are 95% Bonfonerri limits, and inner limits (green) are 95% individual t limits. Values close to/exceeding the inner limits are labelled by pattern. Fig. S4. Prediction expression for SL production with glucose, rapeseed oil and nitrogen (in relation to cornsteep liquor, with a ratio of 1:0.8 cornsteep liquor:ammonium sulfate) for the combined data set from Design 1 and Design 2. Model was sequentially developed from stepwise regression (for model effect combination with the lowest Bayesian Information Criterion value) and standard least squares regression. Fig. S5. Predicted by actual plot of Design 1 (red) and 2 (blue) of the CCD from JMP 15 with the combined regression model. Points labelled by pattern are those that are close to/exceed 95% individual t distribution limits as dictated by externally studentized residuals. Fig. S6. Externally studentized residual plot of results from Design 1 (red) and Design 2 (blue) with the combined regression model. Outer limits (red) are 95% Bonfonerri limits, and inner limits (green) are 95% individual t limits. Values close to/exceeding the inner limits are labelled by pattern. Table S1. Complete data set of executed fermentation flasks under Design 1 and 2 of the [file MBT2-15-1744-s001.docx]

**Microbial Biotechnology**

**Developing an understanding of sophorolipid synthesis through application of a central composite design model**

Benjamin Ingham^a^ and James Winterburn^a,*^

a. Department of Chemical Engineering and Analytical Science, The University of Manchester, Oxford Road, Manchester, M13 9PL, United Kingdom

*Corresponding author

E-mail address: [james.winterburn@manchester.ac.uk](mailto:james.winterburn@manchester.ac.uk)

| **Design** | **Block** | **Flask** | **Pattern** | **Glucose (g/L)** | **Rapeseed oil (mL/L)** | **Nitrogen (g/L)** | **Sophorolipid concentration at 168h (g/L)** | **Predicted Sophorolipid concentration at 168h (g/L) – Design 1 + 2 regression** | **Predicted Sophorolipid concentration at 168h (g/L) – Design 1 regression** |
| --- | --- | --- | --- | --- | --- | --- | --- | --- | --- |
| 1 | 1 | 1 | −−+ | 50 | 50 | 7.5 | 14.25 | 13.362 | 13.439 |
|  |  | 2 | ++− | 150 | 150 | 2.5 | 27.12 | 24.436 | 26.570 |
|  |  | 3 | −++ | 50 | 150 | 7.5 | 18.23 | 13.027 | 13.522 |
|  |  | 4 | −−− | 50 | 50 | 2.5 | 17.67 | 18.939 | 17.999 |
|  |  | 5 | +−+ | 150 | 50 | 7.5 | 14.7 | 14.487 | 13.439 |
|  |  | 6 | −+− | 50 | 150 | 2.5 | 25.85 | 23.311 | 26.570 |
|  |  | 7 | −−− | 50 | 50 | 2.5 | 16.6 | 18.939 | 17.999 |
|  |  | 8 | 000 | 100 | 100 | 5 | 19.63 | 18.973 | 16.409 |
|  |  | 9 | +−− | 150 | 50 | 2.5 | 12.63 | 20.064 | 17.999 |
|  |  | 11 | −++ | 50 | 150 | 7.5 | 11.57 | 13.027 | 13.522 |
|  |  | 12 | +−− | 150 | 50 | 2.5 | 13.15 | 20.064 | 17.999 |
|  |  | 13 | −−+ | 50 | 50 | 7.5 | 12.3 | 13.362 | 13.439 |
|  |  | 14 | +−+ | 150 | 50 | 7.5 | 16.68 | 14.487 | 13.439 |
|  |  | 15 | −+− | 50 | 150 | 2.5 | 22.97 | 23.311 | 26.570 |
|  |  | 16 | +++ | 150 | 150 | 7.5 | 12.23 | 14.152 | 13.522 |
|  |  | 17 | +++ | 150 | 150 | 7.5 | 17.52 | 14.152 | 13.522 |
|  |  | 18 | ++− | 150 | 150 | 2.5 | 29.43 | 24.436 | 26.570 |
|  | 2 | 1 | 00A | 100 | 100 | 9.2 | 15.36 | 17.867 | 19.140 |
|  |  | 2 | 0A0 | 100 | 184.1 | 5 | 16.72 | 20.646 | 17.202 |
|  |  | 3 | 00A | 100 | 100 | 9.2 | 15.82 | 17.867 | 19.140 |
|  |  | 4 | a00 | 15.9 | 100 | 5 | 16.34 | 15.080 | 17.975 |
|  |  | 5 | 00a | 100 | 100 | 0.8 | 39.62 | 35.524 | 33.931 |
|  |  | 6 | a00 | 15.9 | 100 | 5 | 16.58 | 15.080 | 17.975 |
|  |  | 7 | 0A0 | 100 | 184.1 | 5 | 18.92 | 20.646 | 17.202 |
|  |  | 8 | 0a0 | 100 | 15.9 | 5 | 11.06 | 9.417 | 9.924 |
|  |  | 9 | A00 | 184.1 | 100 | 5 | 17.6 | 16.972 | 17.975 |
|  |  | 10 | 000 | 100 | 100 | 5 | 19.06 | 20.435 | 17.975 |
|  |  | 11 | 00a | 100 | 100 | 0.8 | 39.36 | 35.524 | 33.931 |
|  |  | 12 | A00 | 184.1 | 100 | 5 | 19.04 | 16.972 | 17.975 |
|  |  | 13 | 000 | 100 | 100 | 5 | 19.32 | 20.435 | 17.975 |
|  |  | 14 | 0a0 | 100 | 15.9 | 5 | 11.56 | 9.417 | 9.924 |
|  |  | 15 | +−− | 150 | 50 | 2.5 | 20.38 | 21.526 | 19.565 |
|  |  | 16 | ++− | 150 | 150 | 2.5 | 24.98 | 25.899 | 28.136 |
|  |  | 17 | −+− | 50 | 150 | 2.5 | 22.36 | 24.774 | 28.136 |
| 2 | 3 | 1 | 000 | 32.5 | 100 | 0.8 | 34.26 | 32.901 | 33.148 |
|  |  | 2 | +−+ | 50 | 50 | 1.2 | 26.8 | 25.734 | 23.942 |
|  |  | 3 | +−− | 50 | 50 | 0.4 | 35.42 | 29.011 | 27.933 |
|  |  | 4 | −−+ | 15 | 50 | 1.2 | 24.88 | 22.395 | 23.942 |
|  |  | 5 | −−+ | 15 | 50 | 1.2 | 23.78 | 22.395 | 23.942 |
|  |  | 6 | −+− | 15 | 150 | 0.4 | 31.16 | 32.021 | 40.069 |
|  |  | 7 | +−+ | 50 | 50 | 1.2 | 27.38 | 25.734 | 23.942 |
|  |  | 8 | 000 | 32.5 | 100 | 0.8 | 33.54 | 32.901 | 33.148 |
|  |  | 9 | +−− | 50 | 50 | 0.4 | 35.7 | 29.011 | 27.933 |
|  |  | 10 | +++ | 50 | 150 | 1.2 | 29.8 | 31.331 | 34.720 |
|  |  | 11 | ++− | 50 | 150 | 0.4 | 36.6 | 35.361 | 40.069 |
|  |  | 12 | ++− | 50 | 150 | 0.4 | 35.7 | 35.361 | 40.069 |
|  |  | 13 | −++ | 15 | 150 | 1.2 | 26.52 | 27.991 | 34.720 |
|  |  | 14 | +++ | 50 | 150 | 1.2 | 28.9 | 31.331 | 34.720 |
|  |  | 15 | −+− | 15 | 150 | 0.4 | 31.36 | 32.021 | 40.069 |
|  |  | 16 | −−− | 15 | 50 | 0.4 | 28.98 | 25.672 | 27.933 |
|  |  | 17 | −++ | 15 | 150 | 1.2 | 29.76 | 27.991 | 34.720 |
|  |  | 18 | −−− | 15 | 50 | 0.4 | 27.68 | 25.672 | 27.933 |
|  | 4 | 1 | a00 | 3.0686 | 100 | 0.8 | 30.72 | 30.852 | 33.148 |
|  |  | 2 | A00 | 61.9314 | 100 | 0.8 | 39.54 | 36.468 | 33.148 |
|  |  | 3 | 00a | 32.5 | 100 | 0.1273 | 32.06 | 37.780 | 37.294 |
|  |  | 4 | A00 | 61.9314 | 100 | 0.8 | 39.3 | 36.468 | 33.148 |
|  |  | 5 | 00a | 32.5 | 100 | 0.1273 | 31.22 | 37.780 | 37.294 |
|  |  | 6 | a00 | 3.0686 | 100 | 0.8 | 34.14 | 30.852 | 33.148 |
|  |  | 7 | 00A | 32.5 | 100 | 1.4727 | 29.98 | 30.941 | 29.441 |
|  |  | 8 | 0a0 | 32.5 | 15.9 | 0.8 | 12.02 | 13.725 | 19.101 |
|  |  | 9 | 0A0 | 32.5 | 184.1 | 0.8 | 35.48 | 31.606 | 38.372 |
|  |  | 10 | 0A0 | 32.5 | 184.1 | 0.8 | 35.82 | 31.606 | 38.372 |
|  |  | 11 | 00A | 32.5 | 100 | 0.8 | 35.56 | 34.200 | 33.148 |
|  |  | 12 | 0a0 | 32.5 | 15.9 | 0.8 | 14.08 | 13.725 | 19.101 |
|  |  | 13 | 00A | 32.5 | 100 | 1.4727 | 32.56 | 30.941 | 29.441 |
|  |  | 14 | 000 | 32.5 | 100 | 0.8 | 36.64 | 34.200 | 33.148 |
|  |  | 15 | ++0 | 50 | 100 | 0.8 | 37.86 | 35.679 | 33.148 |
|  |  | 16 | ++0 | 50 | 100 | 0.8 | 38.6 | 35.679 | 33.148 |

Supplementary Table 1: Complete data set of executed fermentation flasks under Design 1 and 2 of the central composite circumscribed design. Actual sophorolipid quantities are supplied alongside the predicted values from the regression models of Design 1 and Design 1/2. Nitrogen values refer to the quantity of cornsteep liquor, with a ratio of 1:0.8g/L cornsteep liquor:ammonium sulfate

**Design 1 Initial Regression Model – Supplementary Information**


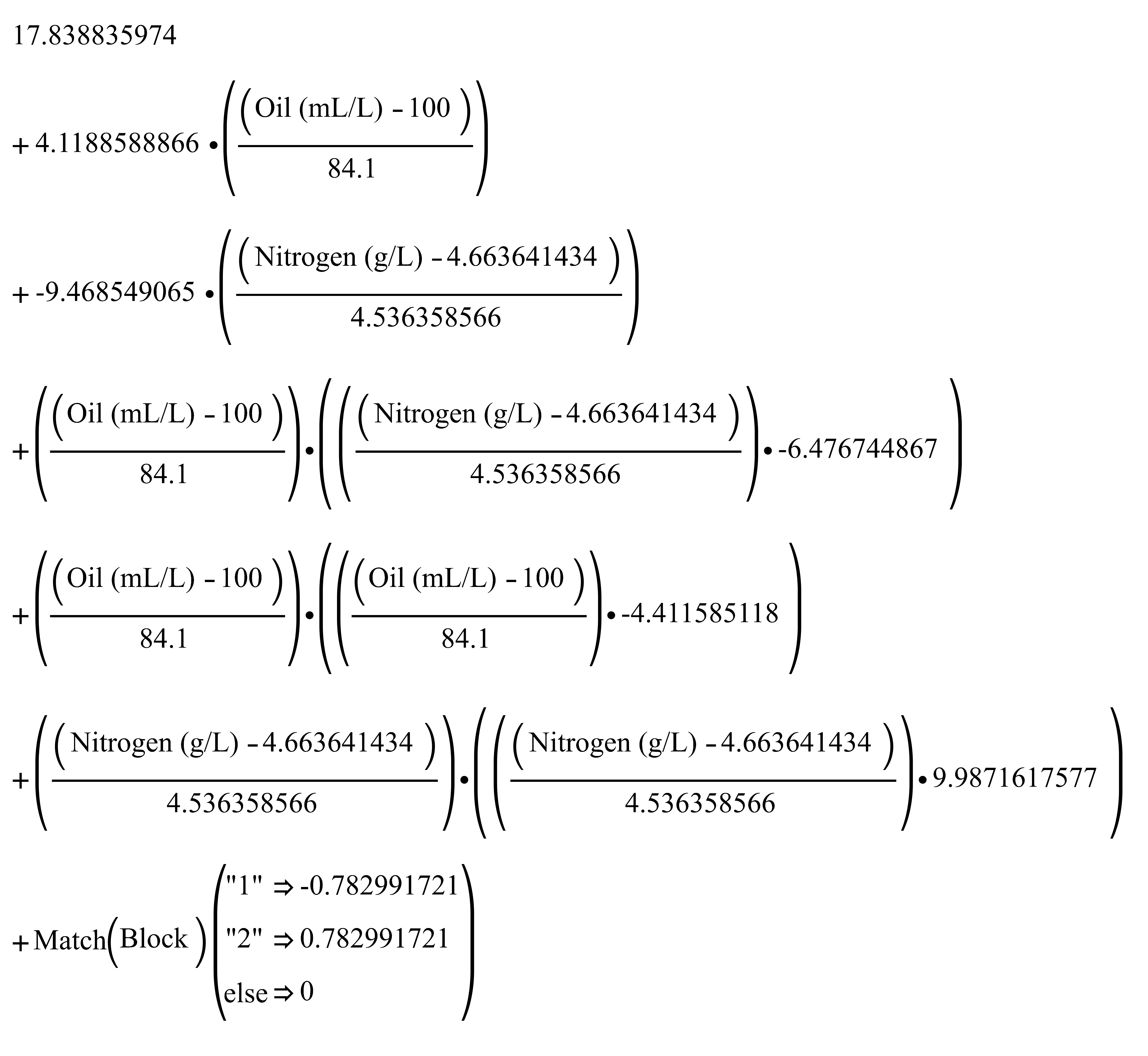


Supplementary Figure 1: Prediction expression for SL production with glucose, rapeseed oil and nitrogen (in relation to cornsteep liquor, with a ratio of 1:0.8g/L cornsteep liquor:ammonium sulfate) for the combined data set from Design 1. Model was sequentially developed from stepwise regression (for model effect combination with the lowest Bayesian Information Criterion value) and standard least squares regression.


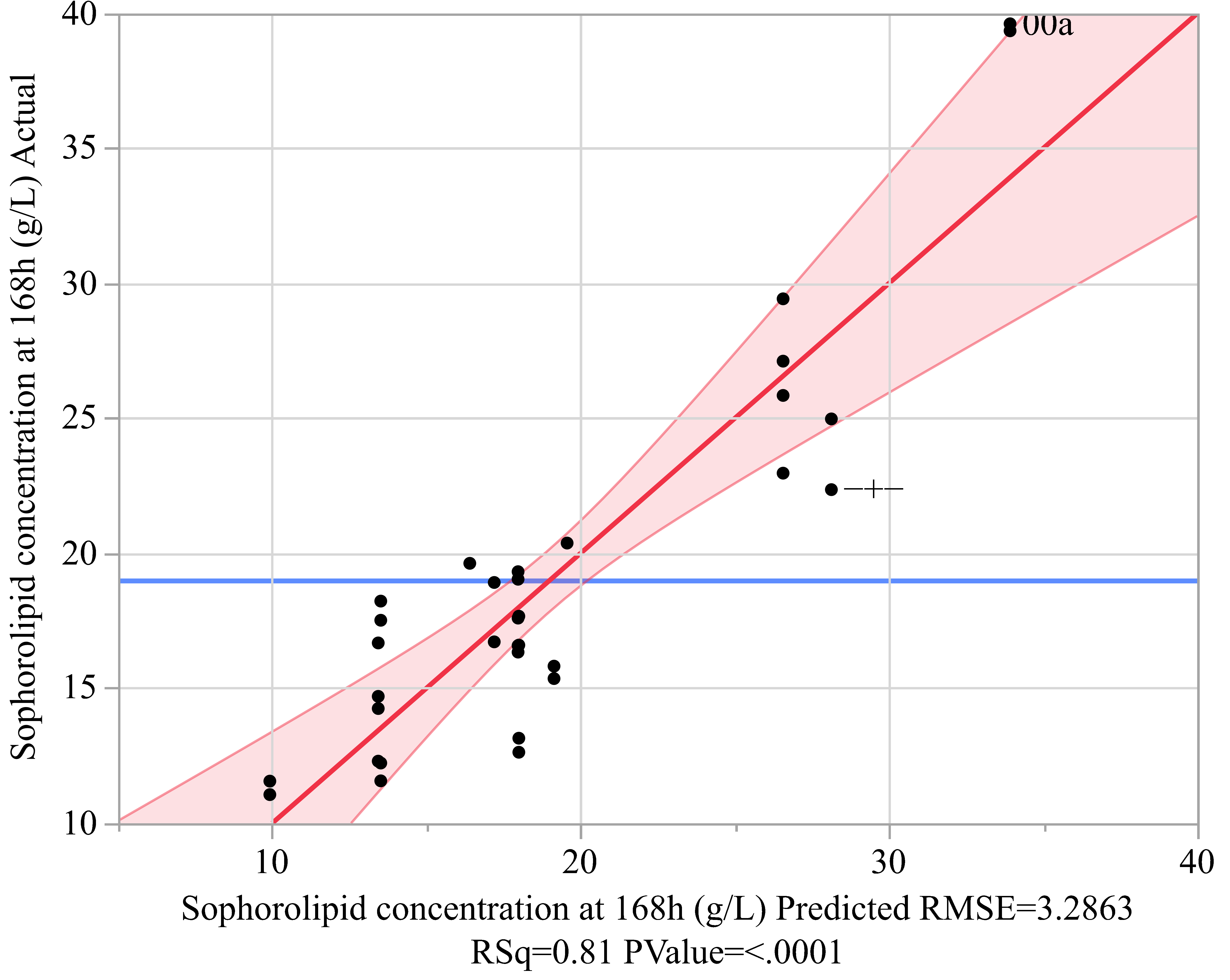


Supplementary Figure 2: Predicted by actual plot of Design 1 values with the initial Design 1 regression model. Points labelled by pattern are those that are close to/exceed 95% individual t distribution limits as dictated by externally studentized residuals.
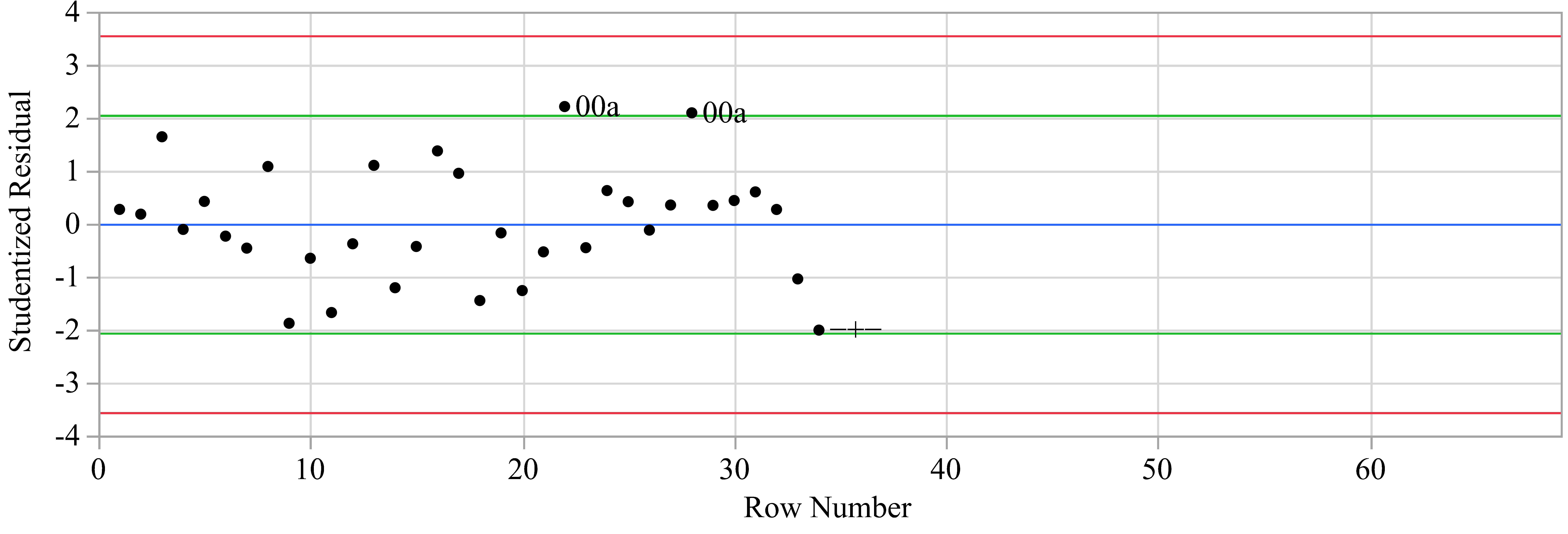


Supplementary Figure 3: Externally studentized residual plot of results from Design 1 with the initial regression model. Outer limits (red) are 95% Bonfonerri limits, and inner limits (green) are 95% individual t limits. Values close to/exceeding the inner limits are labelled by pattern.

**Design 1 and 2 combined regression model – Supplementary Information**





Supplementary Figure 4: Prediction expression for SL production with glucose, rapeseed oil and nitrogen (in relation to cornsteep liquor, with a ratio of 1:0.8 cornsteep liquor:ammonium sulfate) for the combined data set from Design 1 and Design 2. Model was sequentially developed from stepwise regression (for model effect combination with the lowest Bayesian Information Criterion value) and standard least squares regression.

| **Model Effects** | **Number of parameters** | **DF** | **Sum of Squares** | **F Ratio** | **Prob > F** |
| --- | --- | --- | --- | --- | --- |
| Glucose (g/L)(3.06863,184.1) | 1 | 1 | 31.26547 | 3.1708 | 0.0805 |
| Oil (mL/L)(15.9,184.1) | 1 | 1 | 0.12180 | 0.0124 | 0.9119 |
| Nitrogen (g/L)(0.12728, 9.2) | 1 | 1 | 758.36669 | 76.9105 | <.0001* |
| Oil (mL/L)*Nitrogen (g/L) | 1 | 1 | 76.22014 | 7.7299 | 0.0074* |
| Glucose (g/L)*Glucose (g/L) | 1 | 1 | 99.44558 | 10.0854 | 0.0025* |
| Oil (mL/L)*Oil (mL/L) | 1 | 1 | 108.22397 | 10.9756 | 0.0016* |
| Nitrogen (g/L)*Nitrogen (g/L) | 1 | 1 | 129.41209 | 13.1245 | 0.0006* |
| Oil (mL/L)*Oil (mL/L)*Oil (mL/L) | 1 | 1 | 74.43795 | 7.5492 | 0.0081* |
| Oil (mL/L)*Oil (mL/L)*Nitrogen (g/L) | 1 | 1 | 63.19100 | 6.4086 | 0.0142* |
| Block | 3 | 3 | 59.73449 | 2.0193 | 0.1218 |
| Model |  | 12 | 4859.1283 | 41.0661 | <.0001* |
| Error |  | 55 | 542.3207 |  |  |
| Combined total |  | 67 | 5401.4491 |  |  |

Supplementary Table 2: Analysis of variance of the combined regression model and model terms.


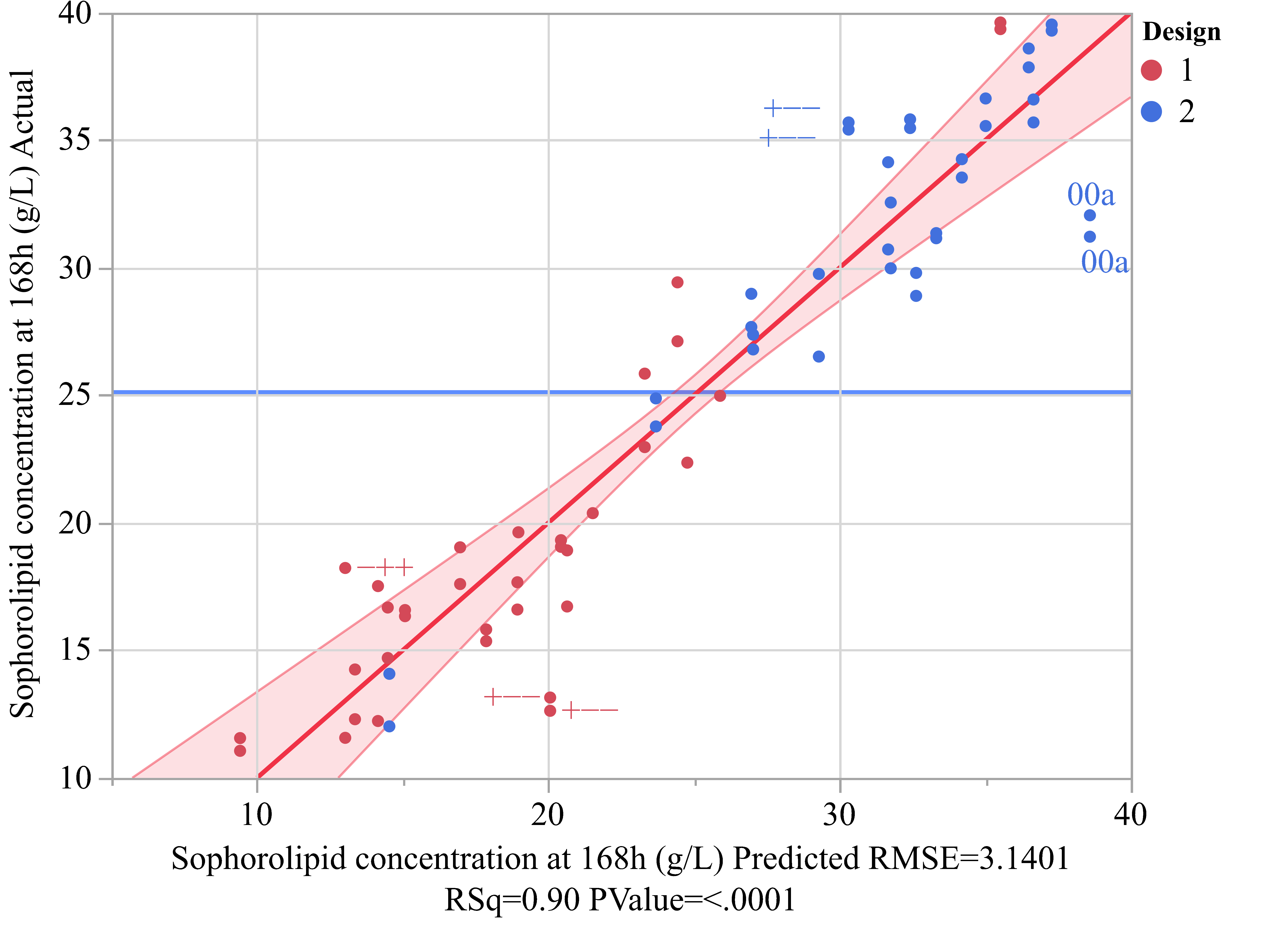


Supplementary Figure 5: Predicted by actual plot of Design 1 (red) and 2 (blue) of the CCD from JMP 15 with the combined regression model. Points labelled by pattern are those that are close to/exceed 95% individual t distribution limits as dictated by externally studentized residuals.


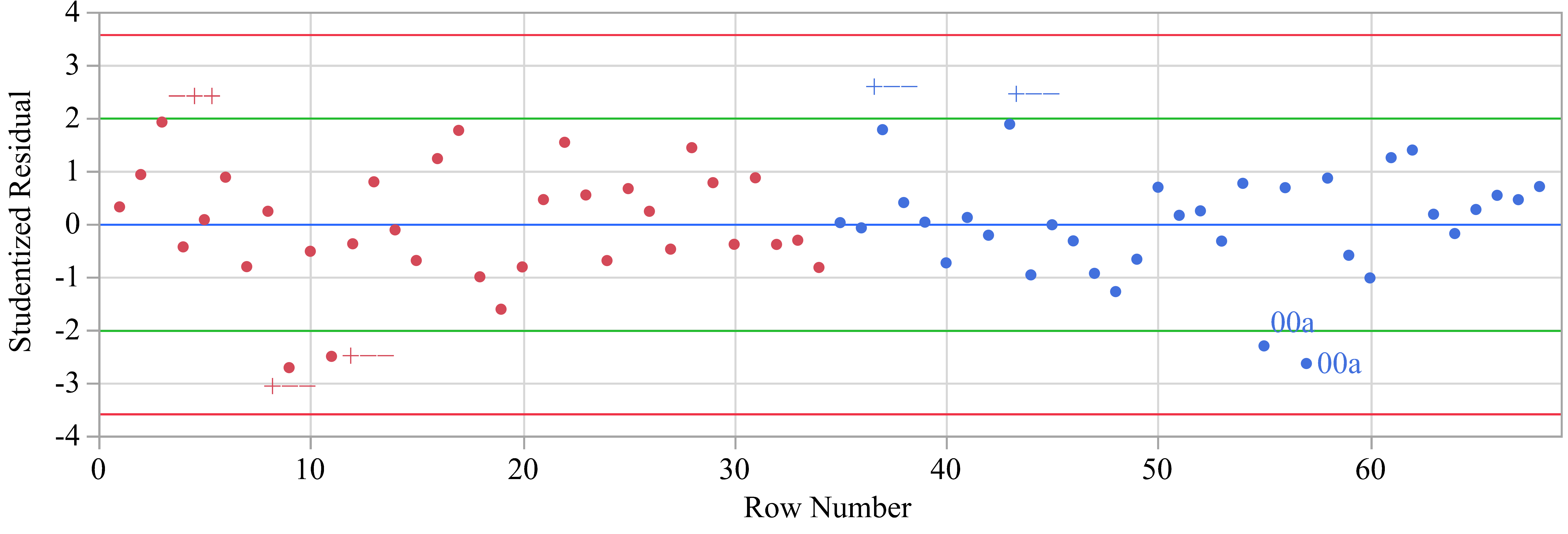


Supplementary Figure 6: Externally studentized residual plot of results from Design 1 (red) and Design 2 (blue) with the combined regression model. Outer limits (red) are 95% Bonfonerri limits, and inner limits (green) are 95% individual t limits. Values close to/exceeding the inner limits are labelled by pattern.
